# Th-POK regulates mammary gland lactation through mTOR-SREBP pathway

**DOI:** 10.1371/journal.pgen.1007211

**Published:** 2018-02-08

**Authors:** Rui Zhang, Huimin Ma, Yuan Gao, Yanjun Wu, Yuemei Qiao, Ajun Geng, Cheguo Cai, Yingying Han, Yi Arial Zeng, Xiaolong Liu, Gaoxiang Ge

**Affiliations:** 1 State Key Laboratory of Cell Biology, CAS Center for Excellence in Molecular Cell Science, Shanghai Institute of Biochemistry and Cell Biology, Chinese Academy of Sciences, Shanghai, People’s Republic of China; 2 CAS Key Laboratory of Systems Biology, CAS Center for Excellence in Molecular Cell Science, Shanghai Institute of Biochemistry and Cell Biology, Chinese Academy of Sciences, Shanghai, People’s Republic of China; 3 University of Chinese Academy of Sciences, Beijing, People’s Republic of China; 4 Innovation Center for Cell Signaling Network, CAS Center for Excellence in Molecular Cell Science, Shanghai Institute of Biochemistry and Cell Biology, Chinese Academy of Sciences, Shanghai, People’s Republic of China; National Cancer Institute, UNITED STATES

## Abstract

The Th-inducing POK (Th-POK, also known as ZBTB7B or cKrox) transcription factor is a key regulator of lineage commitment of immature T cell precursors. It is yet unclear the physiological functions of Th-POK besides helper T cell differentiation. Here we show that Th-POK is restrictedly expressed in the luminal epithelial cells in the mammary glands that is upregulated at late pregnancy and lactation. Lineage restrictedly expressed Th-POK exerts distinct biological functions in the mammary epithelial cells and T cells in a tissue-specific manner. Th-POK is not required for mammary epithelial cell fate determination. Mammary gland morphogenesis in puberty and alveologenesis in pregnancy are phenotypically normal in the Th-POK-deficient mice. However, Th-POK-deficient mice are defective in triggering the onset of lactation upon parturition with large cellular lipid droplets retained within alveolar epithelial cells. As a result, Th-POK knockout mice are unable to efficiently secret milk lipid and to nurse the offspring. Such defect is mainly attributed to the malfunctioned mammary epithelial cells, but not the tissue microenvironment in the Th-POK deficient mice. Th-POK directly regulates expression of insulin receptor substrate-1 (IRS-1) and insulin-induced Akt-mTOR-SREBP signaling. Th-POK deficiency compromises IRS-1 expression and Akt-mTOR-SREBP signaling in the lactating mammary glands. Conversely, insulin induces Th-POK expression. Thus, Th-POK functions as an important feed-forward regulator of insulin signaling in mammary gland lactation.

## Author summary

Th-POK, aka cKrox or ZBTB7B, is a zinc finger transcription factor that specifies the cell fate of immature T cell precursors towards the CD4 lineage. It is yet largely unknown if Th-POK participates in the regulation of other biological processes. It is also not known if Th-POK functions beyond cell fate determination. In this study, we found that Th-POK is expressed in the luminal, but not the basal epithelial cells in the mammary glands. Despite the lineage restricted expression in the mammary glands, Th-POK is dispensable for mammary epithelial cell lineage specification and mammary gland development. Rather, Th-POK regulates the onset of lactation and milk lipid production via mammary epithelial cell autonomous mechanisms, suggesting that Th-POK exerts distinct functions in a tissue specific manner. Th-POK regulates the expression of insulin receptor sunstrate-1 and insulin-induced mTOR-SREBP pathway activation and lipid biosynthesis in the mammary alveolar cells at lactation. Thus, Th-POK functions as an important metabolic regulator in the lactating mammary glands, in addition to its well documented cell fate specification functions in T cell development.

## Introduction

Transcription factor Th-POK (Th-inducing POK, also known as ZBTB7B or cKrox) is a key regulator of lineage commitment of immature T cell precursors [1–3]. Loss of Th-POK expression or impaired Th-POK function disrupts the development of CD4 T cells [4], whereas enforced expression of Th-POK results in lineage conversion to the CD4 fate [4–6]. Th-POK is positively regulated by GATA-3, a transcription factor essential for CD4 fate determination [6, 7], and negatively regulated by CD8 lineage-specific transcription factor Runx3 [8]. Th-POK expression is also regulated post-transcriptionally by p300-mediated acetylation [9]. Th-POK acts as both transcriptional repressor and activator to regulate gene expression [1, 10, 11]. Cross-antagonism between Th-POK and Runx3 are determinative to CD4 versus CD8 cell fate decision. It is yet largely unknown the physiological functions of Th-POK besides helper T cell differentiation.

Mammary glands develop from a rudimentary tree to a branched epithelial network of ducts during puberty that further undergo alveologenesis to create a lactation-competent gland upon pregnancy [12–14]. Morphogenesis and differentiation of the mammary gland to a functional milk-producing organ are precisely regulated [12–14]. GATA-3, the critical transcription factor in T cell CD4 fate determination upstream of Th-POK, is the most highly enriched transcription factor in the mammary epithelium of pubertal mice [15]. GATA-3 is critical to the maintenance of proper luminal progenitor pool at puberty and alveolar differentiation during pregnancy [15, 16].

Upon parturition, milk protein and lipid biosynthesis capability in the alveolar epithelial cells are sharply increased [12, 14, 17]. The key event in the onset of milk secretion is the release of cytosolic lipid droplets (CLDs) by the alveolar epithelial cells into the luminal space [12, 14, 17]. Reduced milk lipid production and secretion in lactating mammary glands have been linked to poor newborn survival [18–25]. Interaction between xanthine oxidoreductase (XOR) and butyrophilin (BTN) is essential to the secretion of CLDs [21, 23, 26]. Proteins regulating XOR expression, e.g. Cidea [24], are important regulators of the CLD secretion. Lipid biosynthesis are tightly regulated at transition from pregnancy to lactation [17, 27]. Insulin signaling through its receptor (IR) is a potent regulator of cellular metabolism. Deletion of IR in the mammary epithelial cells at pregnancy resulted in reduced milk protein and lipid production [28]. Akt1 downstream of IR is required for the upregulated lipid synthesis in the lactating mammary glands. Akt1 deficiency resulted in impaired lipid biosynthesis in the lactating mammary gland [19]. Ectopic expression of constitutively active Akt in the mammary glands led to excess lipid synthesis during pregnancy and lactation [29]. Prolactin is demonstrated to regulate a subset of mammary epithelial cell specific lipogenic gene expression in lactating mammary glands [30]. Mice deficient of Src, a regulator of the prolactin signaling, or its binding protein actin filament-associated protein 1 (AFAP1) exhibited retention of large CLDs [20, 25]. Sterol regulatory element binding proteins (SREBPs), the key transcription factors regulating fatty acid and cholesterol biosynthesis [31–33], are proposed as key regulators in efficient lipid synthesis at the onset of milk secretion [17, 20, 27, 34]. Akt-mTOR signaling is required for insulin-induced SREBP1 expression and processing [35–37]. Akt regulates lipid biosynthesis via SREBP1 [37]. AFAP1 deficiency reduced SREBP1 levels in the lactating mammary glands [20].

In this study, we identified Th-POK as a luminal-specific expressing transcription factor in the mammary glands. Knockout of Th-POK did not affect pubertal growth or alveologenesis of mammary gland. However, the transition from pregnancy to lactation upon parturition was compromised in the Th-POK knockout mice. Th-POK deficiency impaired insulin-induced activation of mTOR-SREBP pathway and lipid biosynthesis. As a result, Th-POK knockout mice were unable to efficiently secret milk and to nurse the offspring. Thus, Th-POK functions as a critical regulator in mammary gland lactation.

## Results

### Th-POK is expressed in mammary luminal epithelial cells and required for pup survival

**Fig 1:**
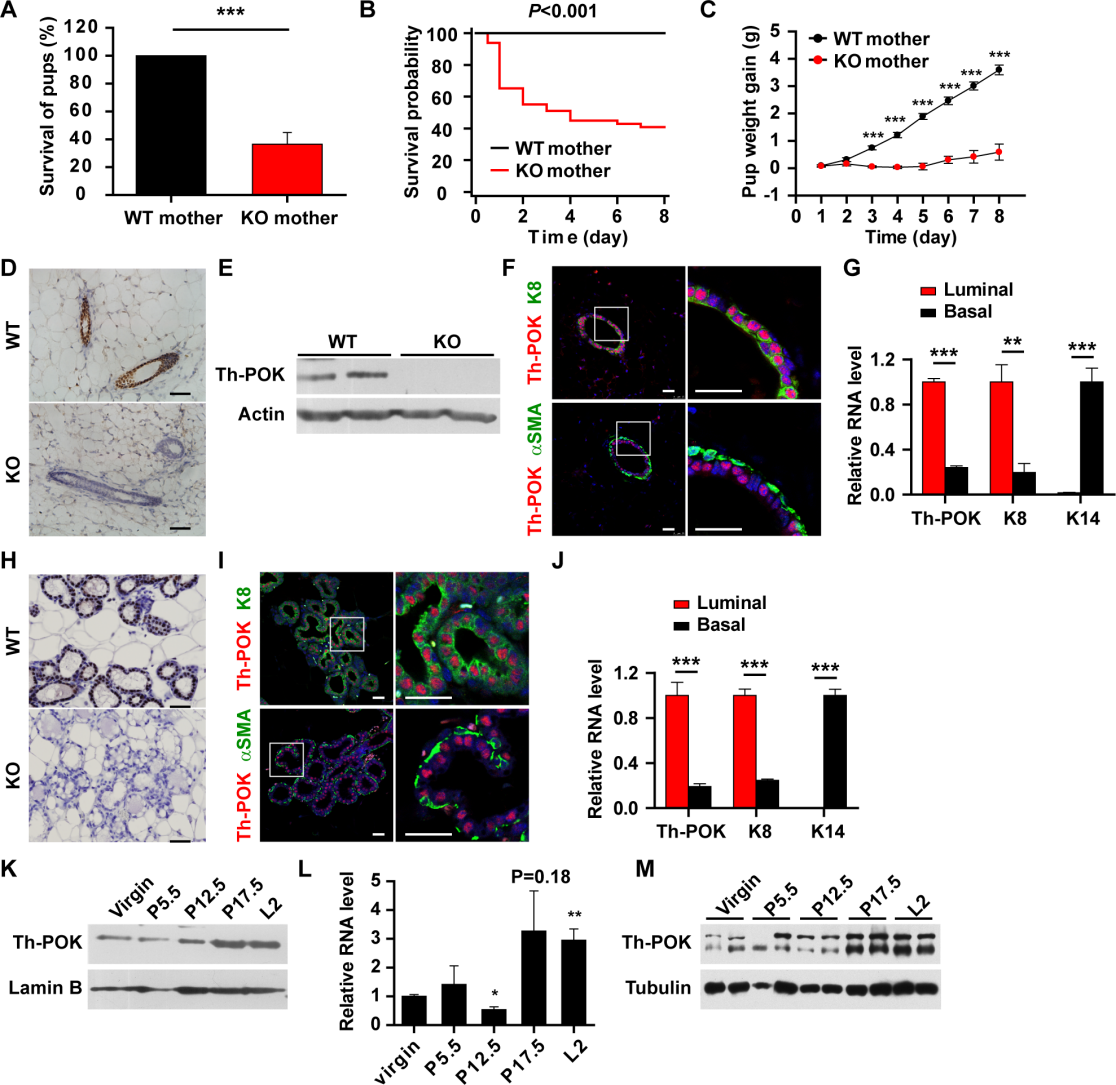
Th-POK is expressed in the mammary luminal epithelial cells and is essential to pup survival. (A) Wild-type (WT) females were mated with Th-POK knockout (KO) males and KO females were mated with WT males. Percent of survived pups nursed by either WT or KO dams (N = 30) within 48 hours after birth. (B) Heterozygote pups born to WT females were fostered to WT (N = 7) or KO (N = 7) dams at the time of parturition. Kaplan-Meier survival analysis of the pups. (C) Weight gain of the pups fostered to WT (N = 7) or KO (N = 5) dams. (D) Immunostaining of Th-POK on mammary gland sections from WT and KO virgin mice. Scale bar: 50μm. (E) Western blot analysis of Th-POK protein levels in isolated mammary epithelial cells from WT and KO virgin mice. (F) Double staining of Th-POK (red) with luminal cell marker cytokeratin 8 (K8) or basal cell marker αSMA (green) on virgin mammary gland sections. Scale bars: 25μm. (G) RT-qPCR analyses of expression of Th-POK, luminal cell marker cytokeratin 8 (K8) and basal cell marker cytokeratin 14 (K14) in luminal (Lin^-^, CD24^+^, CD29^lo^) and basal (Lin^-^, CD24^+^, CD29^hi^) mammary epithelial cells FACS-isolated from virgin mice (N = 4). (H) Immunostaining of Th-POK on mammary gland sections from WT and KO mice at lactation day 2 (L2). Scale bar: 50μm. (I) Double staining of Th-POK (red) with luminal cell marker cytokeratin 8 (K8) or basal cell marker αSMA (green) on mammary gland sections at L2. Scale bars: 25μm. (J) RT-qPCR analyses of expression of Th-POK, K8 and K14 in luminal and basal mammary epithelial cells FACS-isolated from mice at L2 (N = 4). Data are presented as mean ± SEM. **P < 0.01, ***P < 0.001. (K) Western blot analysis of Th-POK expression in mammary glands at different stages. (L and M) RT-qPCR (L, N = 3) and western blot (M) analyses of Th-POK expression in isolated mammary epithelial cells at different stages. Data are presented as mean ± SEM. *P < 0.05, **P < 0.01, compared to virgin.

In an attempt to breed with Th-POK knockout (KO) mice, wild-type (WT) females were mated with KO males and KO females were mated with WT males. All (30/30) KO males were competent in producing healthy offspring (Fig 1A). However, significantly decreased survival of pups born to KO females was observed (Fig 1A). This was not due to the pup genotype, as all the pups were heterozygous for Th-POK. To investigate if the observed defect in pup viability born to KO mice was dependent on the genotype of the mother, pups born to WT females were fostered to KO mice. ~50% of the pups died during the course of study (Fig 1B). The surviving pups exhibited reduced growth, compared to the pups nursed by WT mothers (Fig 1C). Thus, Th-POK is required for lactating mice to support the survival and growth of their litters.

GATA-3, a transcription factor upstream of Th-POK in T cell development, is the most highly enriched transcription factor in the mammary epithelium of pubertal mice and a critical regulator of luminal differentiation [15, 16]. The inability of KO mice to properly nurse their pups promoted us to study if Th-POK is expressed in the mammary gland and plays a role in mammary gland development and function. Immunohistochemical staining on mammary gland sections showed that Th-POK was expressed in mammary epithelial cells of virgin mice (Fig 1D). Western blot analysis further confirmed that Th-POK protein was expressed in the mammary epithelial cells isolated from the mammary glands of virgin mice (Fig 1E). The mammary gland is composed of basal layer myoepithelial cells and inner layer luminal cells [13, 38, 39]. Th-POK colocalized with luminal marker cytokeratin 8 (K8), but not basal marker α-smooth muscle actin (αSMA) (Fig 1F). Th-POK mRNA levels were significantly higher in the K8-positive luminal cells than in the K14-positive basal cells (Fig 1G). Thus, Th-POK is expressed restrictedly in the luminal lineage. At lactation, Th-POK was expressed in the luminal epithelial cells of alveoli (Fig 1H–1J). Analysis of Th-POK expression at different mammary developmental stages revealed that its expression levels were upregulated at late pregnancy (day 17.5) and remained high at the lactation stage (Fig 1K and S1 Fig). Analyses of Th-POK expression in the isolated mammary epithelial cells further revealed increased Th-POK mRNA and protein levels at late pregnancy and lactation (Fig 1L and 1M).

### Normal mammary morphogenesis and secretory differentiation in Th-POK-deficient mice

**Fig 2:**
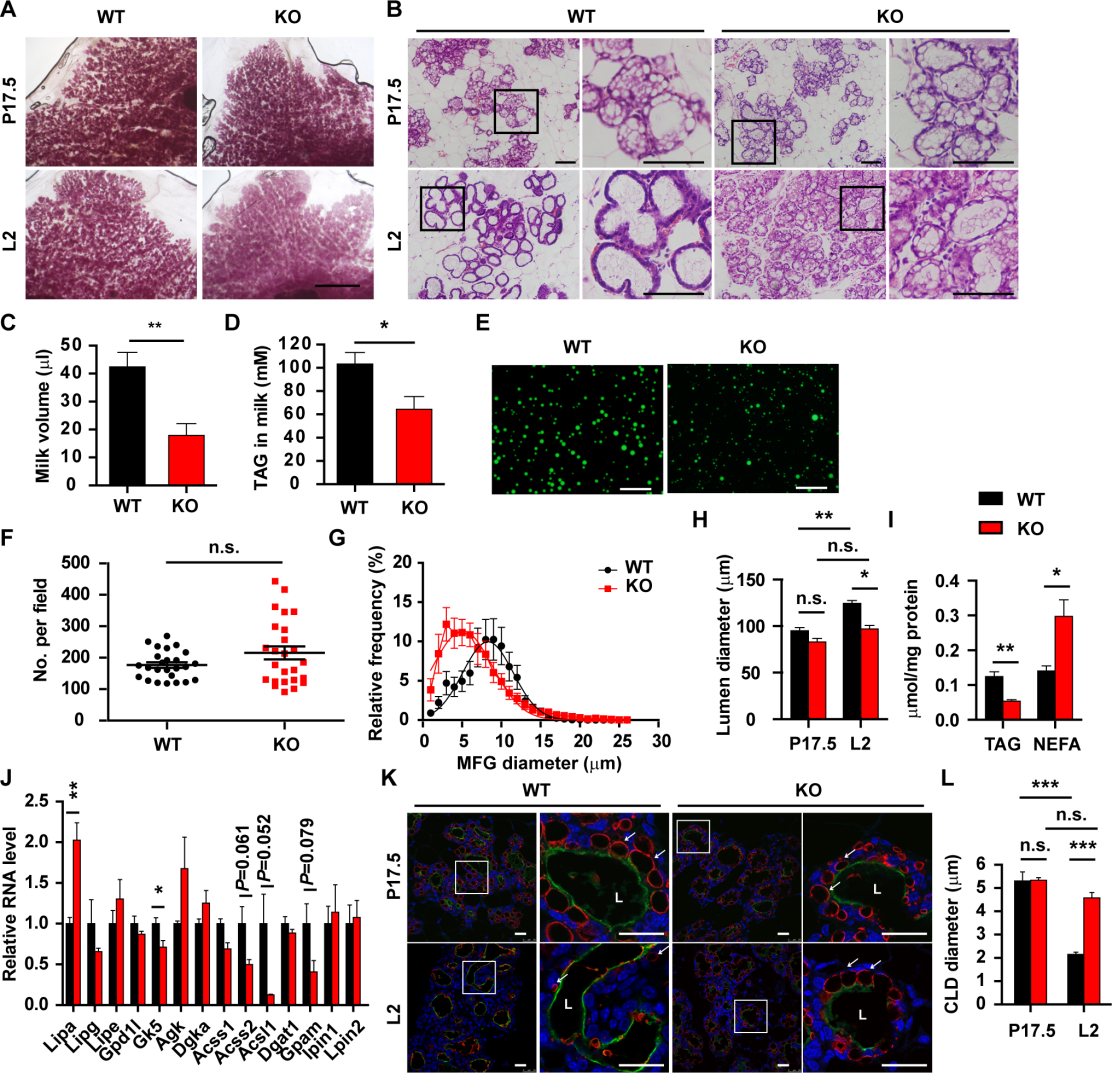
Impaired milk lipid secretion in Th-POK-deficient mammary glands. (A) Carmine-stained whole-mounted mammary glands from WT and KO mice at pregnancy day 17.5 (P17.5) or lactation day 2 (L2). Scale bar: 2mm. (B) Hematoxylin-and-eosin-stained sections of mammary glands from WT and KO mice at P17.5 or L2. Scale bar: 100μm. (C and D) Milk was collected from fourth mammary glands following oxytocin stimulation at lactation day 2. Milk volume (WT = 11, KO = 8) (C) and milk triacylglycerol (TAG) concentration (WT = 10, KO = 6) (D) were compared. (E) Staining of MFGs from WT and KO mice. Scale bars: 100μm. (F) Number of MFGs from WT and KO mice (N = 5, five fields/mice). (G) Size distribution of MFGs from WT and KO mice (N = 5). (H) Lumen diameters of mammary glands from WT and KO mice at P17.5 or L2 (N = 3, five fields/mice). (I) Triacylglycerol (TAG) and nonesterified fatty acid (NEFA) concentrations in the mammary epithelial cells at L2 (WT = 4, KO = 4). (J) RT-qPCR analyses of genes regulating lipolysis and triacylglycerol synthesis in mammary glands (N = 4 mammary glands from individual mice) at lactation day 2. (K) Sections of mammary glands from WT and KO mice at P17.5 or L2 were stained with anti-Perilipin2 (red). The luminal surface of secretory alveoli was visualized with wheat germ agglutinin (green). CLDs are marked by arrows. Scale bars: 25μm. (L) Quantification of CLD size in mammary glands from WT and KO mice at P17.5 or L2 (N = 3, five fields/mice). Data are presented as mean ± SEM. Statistic analyses were performed with two-way Anova followed by Bonferroni's multiple comparison test (H and K). *P < 0.05, **P < 0.01, ***P < 0.001. n.s.: not significant.

As Th-POK is specifically expressed in luminal epithelial cells, we next examined if Th-POK deficiency would affect mammary gland development in a manner similar to GATA-3. As shown by whole-mount analyses, comparable ductal outgrowth and numbers of terminal end buds were noticed in WT and KO mammary glands from virgin mice (S2A–S2C Fig). Histology inspection showed indistinguishable architecture of terminal end buds at 5 weeks and mammary ducts at 7 and 10 weeks between WT and KO mice (S2D Fig). Mammary epithelial compartment undergoes lobuloalveolar development in pregnancy to form the alveolar secretory units [13, 38, 39]. Mammary glands from the KO mice at early-, mid- to late-term pregnant mice (postcoital days 5.5, 12.5 and 17.5, respectively) showed normal organization of lobuloalveolar structures (S2E and S2F Fig, Fig 2A and 2B). Expansion of the mammary epithelium is achieved by active cell proliferation. BrdU incorporation analyses revealed comparable rates of cell proliferation in the mammary glands from WT and KO mice (S2G and S2H Fig). The mammary epithelial cells differentiate into functional alveolar cells during pregnancy. Such secretory differentiation process is characterized by upregulation of milk protein expression [12]. mRNA levels of milk proteins were indistinguishable between WT and KO mammary glands (S3A Fig). Thus, Th-POK deficiency does not affect mammary epithelial cell fate determination and mammary gland development at puberty and pregnancy.

### Impaired milk lipid production and secretion in Th-POK-deficient mammary glands

The increased mortality and growth retardation in pups nursed by the KO mice suggested that the KO mice may be defective in milk production at lactation stage. Indeed, lactating KO mice had 2.2-fold reduction in oxytocin-stimulated milk secretion compared to WT mice (Fig 2C). Protein and lipid are the major components of milk [12, 14]. Milk protein concentration and composition from the lactating KO mice were largely similar to that from the WT mice (S3B and S3C Fig). This promoted us to investigate whether lipid production and secretion were compromised in the lactating KO mice. The concentration of triacylglycerol (TAG), the main component of the milk lipid [12, 17], was markedly lower in milk taken from lactating KO mice compared with WT mice (Fig 2D). Milk lipids are secreted as milk fat globules (MFGs), with lipid droplets wrapped by a plasma membrane bilayer. Although the numbers of MFGs were similar in the milk from lactating WT and KO mice (Fig 2E and 2F), MFGs from the lactating KO mice were substantially smaller in size compared to those from lactating WT mice (Fig 2E and 2G). At parturition, release of CLDs from the alveolar epithelial cells is a critical event in producing sufficient milk lipid [12, 14, 17]. At late-pregnancy (P17.5), large CLDs accumulated in the alveolar epithelial cells in both WT and KO mammary glands (Fig 2B). Following parturition, large CLDs were replaced by small lipid droplets in alveolar epithelial cells in the WT mammary glands at lactation day 2 (L2) (Fig 2B). In contrast to that in the WT mammary glands, luminal space was much less expanded in the KO mammary glands (Fig 2B and 2H). Large CLDs were retained within alveolar epithelial cells in the KO mammary glands after parturition (Fig 2B), suggesting impaired milk lipid secretion in the KO mice. Cytosolic TAG concentration was significantly lower, whereas nonesterified fatty acid (NEFA) concentration was significantly higher in KO mammary epithelial cells than in WT mammary epithelial cells at L2 (Fig 2I). Expression of genes invloved in lipolysis and TAG synthesis was substantially altered in the KO mammary epithelial cells at L2 (Fig 2J). To further confirm the retention of large CLDs in the KO alveolar epithelial cells and failure in lipid secretion, sections of mammary glands were stained for Perilipin2 (Plin2, also known as adipophilin), a lipid droplet surface protein present in the CLDs in the alveolar epithelial cells [40] (Fig 2K). At late-pregnancy (P17.5), large CLDs were present in alveolar epithelial cells in both WT and KO mammary glands with similar size (Fig 2K and 2L). On lactation day 2, only small size CLDs were present at the apical surface of the WT alveolar epithelial cells, whereas large amount of large size CLDs were apparent in the KO alveolar epithelial cells (Fig 2K and 2L).

### Precocious involution in Th-POK knockout mammary glands

**Fig 3:**
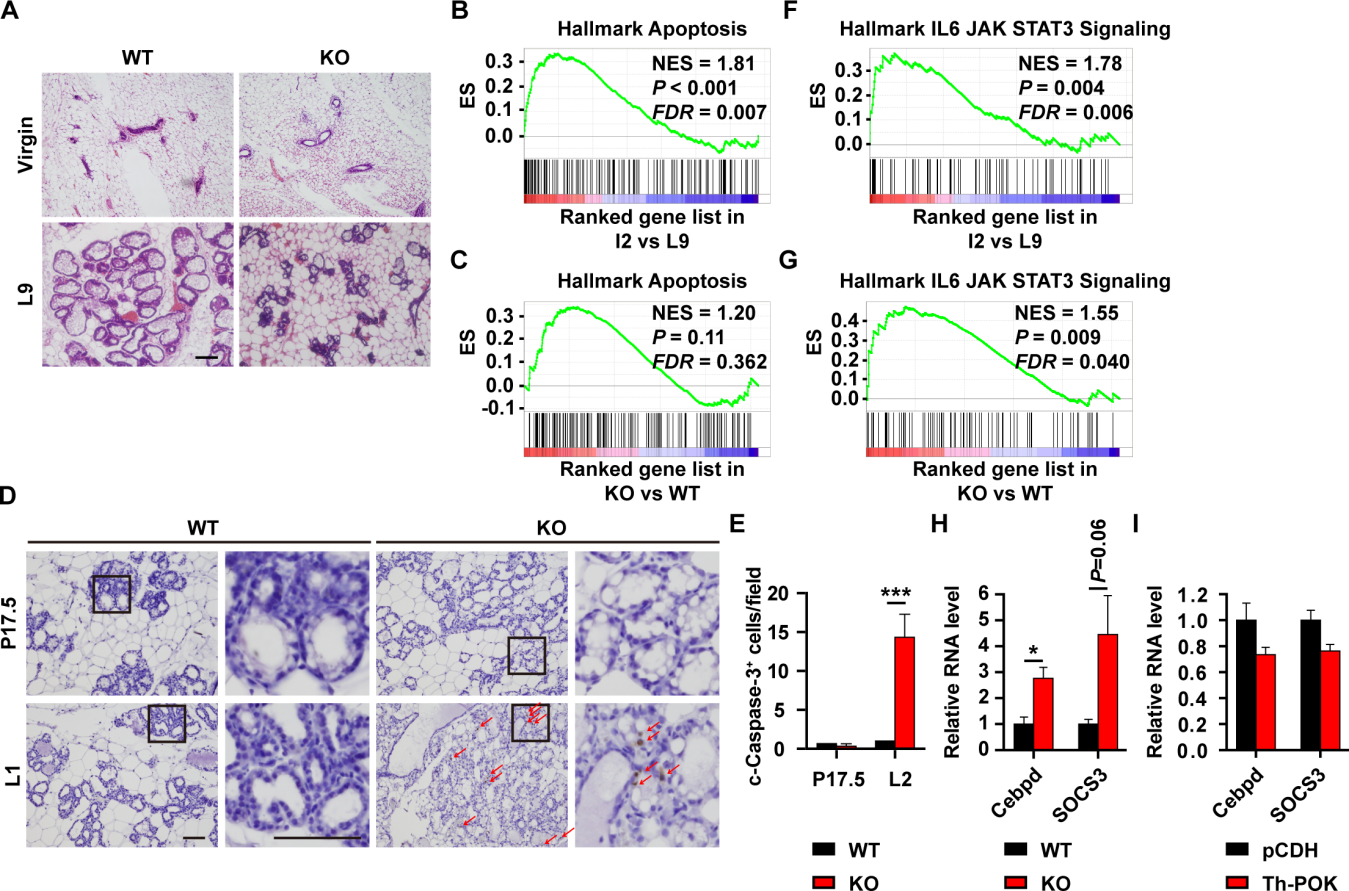
Precocious involution in Th-POK knockout mammary glands. (A) Hematoxylin-and-eosin-stained sections of mammary glands from 12-week virgin mice and mice at lactation day 9. Scale bar: 100μm. (B) GSEA data showing the enrichment of apoptosis signature in mammary glands at involution day 2, compared to those at lactation day 9. (C) GSEA data showing marginal enrichment of apoptosis signature in KO mammary gland compared to the WT mammary gland at lactation day 1. (D) Immunostaining of cleaved caspase-3 on mammary gland sections from WT and KO mice at pregnancy day 17.5 (P17.5) and lactation day 1 (L1). Scale bar: 50μm. (E) Quantitative analysis of cleaved caspase-3 staining in (D). Data are represented as the number of cleaved caspase-3-positive cells per field (N = 3, five 20× fields/mice). (F) GSEA data showing the enrichment of IL6-JAK-STAT3 signature in mammary glands at involution day 2, compared to those at lactation day 9. (G) GSEA data showing enrichment of IL6-JAK-STAT3 signature in KO mammary gland compared to the WT mammary gland at lactation day 1. (H) RT-qPCR analyses of expression of *Cebpd* and *Socs3* in mammary glands from WT and KO mice at lactation day 2 (N = 4). (I) RT-qPCR analyses of expression of *Cebpd* and *Socs3* in Th-POK-expressing HC11 cells. Data are presented as mean ± SEM. *P < 0.05, ***P < 0.001. ES: Enrichment score. NES: normalized enrichment score. *FDR*: false discovery rate.

Upon weaning of the pups, the mammary gland undergoes involution, a process characterized by the apoptosis of the mammary epithelial cells and remodeling of the mammary gland back to a virgin-like morphology. Precocious involution was frequently observed in mouse models that display defects in secretory activation [20, 21, 23, 25]. At lactating day 9, mammary glands from 50% of the KO mice displayed morphology similar to virgin mice, suggesting precocious involution had occurred (Fig 3A). Genes involved in cell apoptosis [41] were enriched in the WT mammary gland at involution stage (Fig 3B). In the mammary glands of lactating KO mice, the apoptosis signature was marginally enriched (Fig 3C). Cleaved caspase-3 signal was detected in the KO alveoli, but not in the WT alveoli at lactation (Fig 3D and 3E). IL-6-triggered activation of JAK-Stat3 pathway is the key modulator of apoptosis during mammary gland involution [42, 43]. IL-6-JAK-STAT3 pathway [41] was enriched in the mammary gland at involution stage (Fig 3F), and in the lactating KO mammary glands (Fig 3G). Expression of *Cebpd* and *Socs3*, genes that are specifically expressed at the involution stage [42–44], was significantly upregulated in the lactating KO mammary glands (Fig 3H). Expression of Th-POK in HC11 mammary epithelial cells did not affect expression of *Cebpd* and *Socs3* (Fig 3I), suggesting Th-POK did not proactively regulate *Cebpd* and *Socs3* expression and the premature involution program. Indeed, cleaved caspase-3 signal was not detected in the KO alveoli at late pregnancy (Fig 3D and 3E).

### Defective lactation is intrinsic to mammary epithelial cells

**Fig 4:**
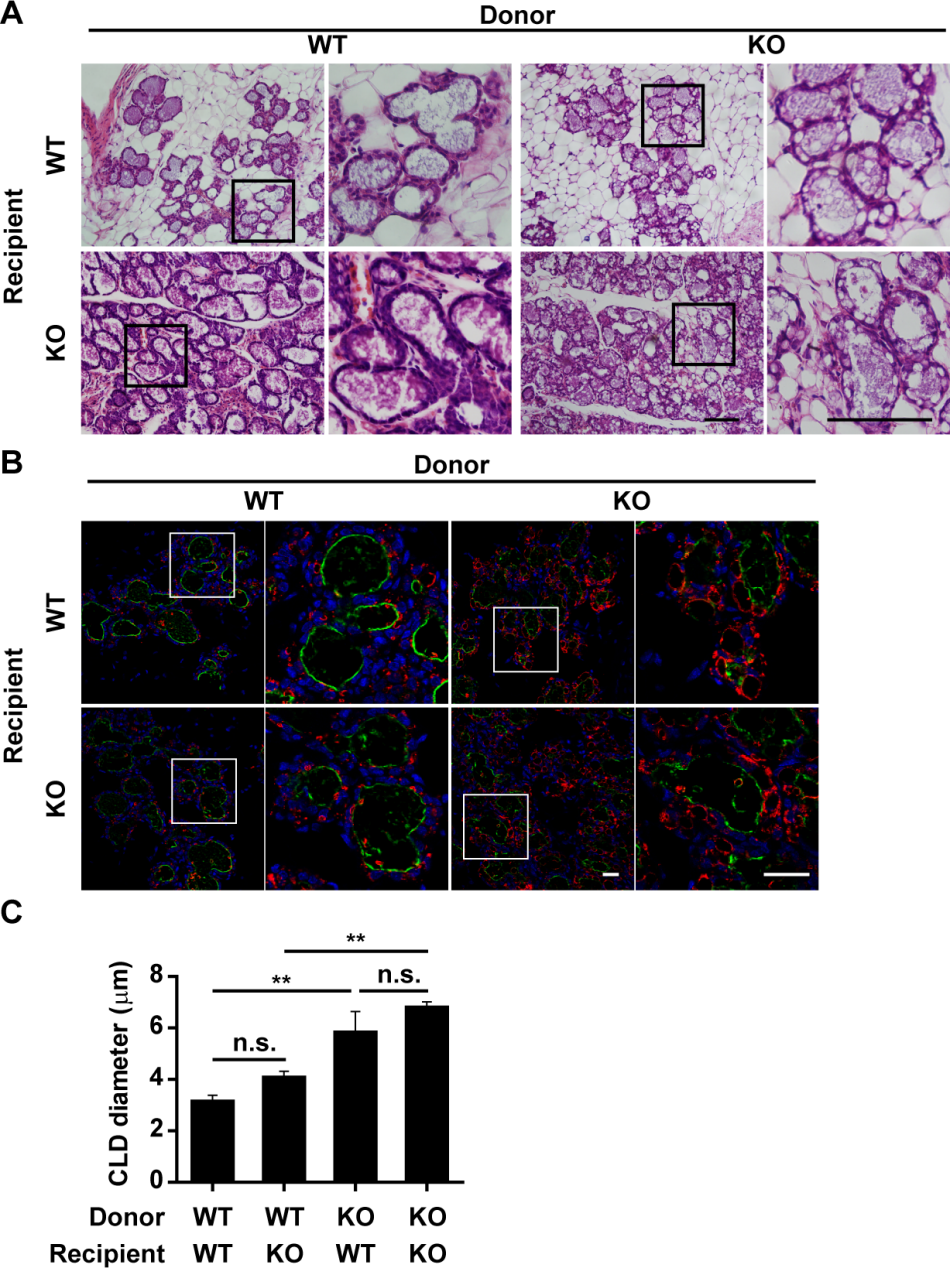
Impaired lipid secretion in Th-POK-deficient mammary glands is attributed mainly to intrinsic defects in the mammary epithelial cells. Wild-type or Th-POK-deficient mammary epithelial cells were implanted into clear fat pads of wild-type or Th-POK-deficient mice. Reconstituted mammary glands were harvested at lactation day 2. (A) Hematoxylin-and-eosin-stained sections of reconstituted mammary glands at lactation day 2. Scale bar: 100μm. (B) Sections of reconstituted mammary glands at lactation day 2 were stained with anti-Perilipin2 (red). The luminal surface of secretory alveoli was visualized with wheat germ agglutinin (green). Scale bars: 25μm. (C) Quantification of CLD size in transplanted mammary glands from WT and KO mice (N = 3, five fields/mice). Data are presented as mean ± SEM. Statistic analyses were performed with two-way Anova followed by Bonferroni's multiple comparison test. **P < 0.01. n.s.: not significant.

Th-POK deficiency disrupts the development of CD4 T cells [4]. To investigate if the failure in milk lipid secretion is due to altered immune environment, mammary epithelial cells isolated from wild-type or Th-POK-deficient mammary glands were implanted into the cleared fat pads of wild-type or Th-POK KO mice (Fig 4). Both wild-type and Th-POK-deficient mammary epithelial cells developed into properly structured mammary glands, further reinforcing the notion that Th-POK is not required for mammary gland development (Fig 4A). Mammary glands developed from the wild-type mammary epithelial cells successfully released lipid into the luminal space with small lipid droplets retained in the mammary epithelial cells on lactation day 2, regardless the genotype of the recipient mice (Fig 4). However, the mammary glands developed from the Th-POK-deficient mammary epithelial cells retained large size lipid droplets within the mammary epithelial cells upon parturition in both wild-type and Th-POK-deficient recipient mice (Fig 4). These data collectively suggested that the defective lipid secretion phenotype observed in the Th-POK-deficient mice was mainly due to the defects in the mammary epithelial cells.

### Th-POK deficiency impairs SREBP activity in lactating mammary glands

Cell polarity is prerequisite for milk secretion [39]. The apical marker Ezrin and basolacteral marker E-cadherin were properly expressed and localized in the KO alveolar epithelial cells (S4A Fig). Mice deficient of BTN [21] or heterozygous for XOR [23] had defective lipid droplet secretion and accumulated large lipid droplets in the cytoplasm of mammary epithelial cells after parturition. Expression of BTN and XOR or the XOR reductase activity was indistinguishable between WT and KO mammary glands (S4B–S4D Fig). Expression of lipid droplet surface protein Plin2 or Cidea was also unchanged between WT and KO mammary glands (S4B Fig) [24, 40]. Mice deficient of Src or its binding protein AFAP1 exhibit retention of large CLDs [20, 25]. However, there was no significant difference in Src signature [45] (NES = -1.09, *P* = 0.285) between WT and KO mammary glands (S4E Fig). Western blot analyses also revealed that neither Src expression nor its phosphorylation was disturbed in the lactating KO mammary glands (S4C Fig).

**Fig 5:**
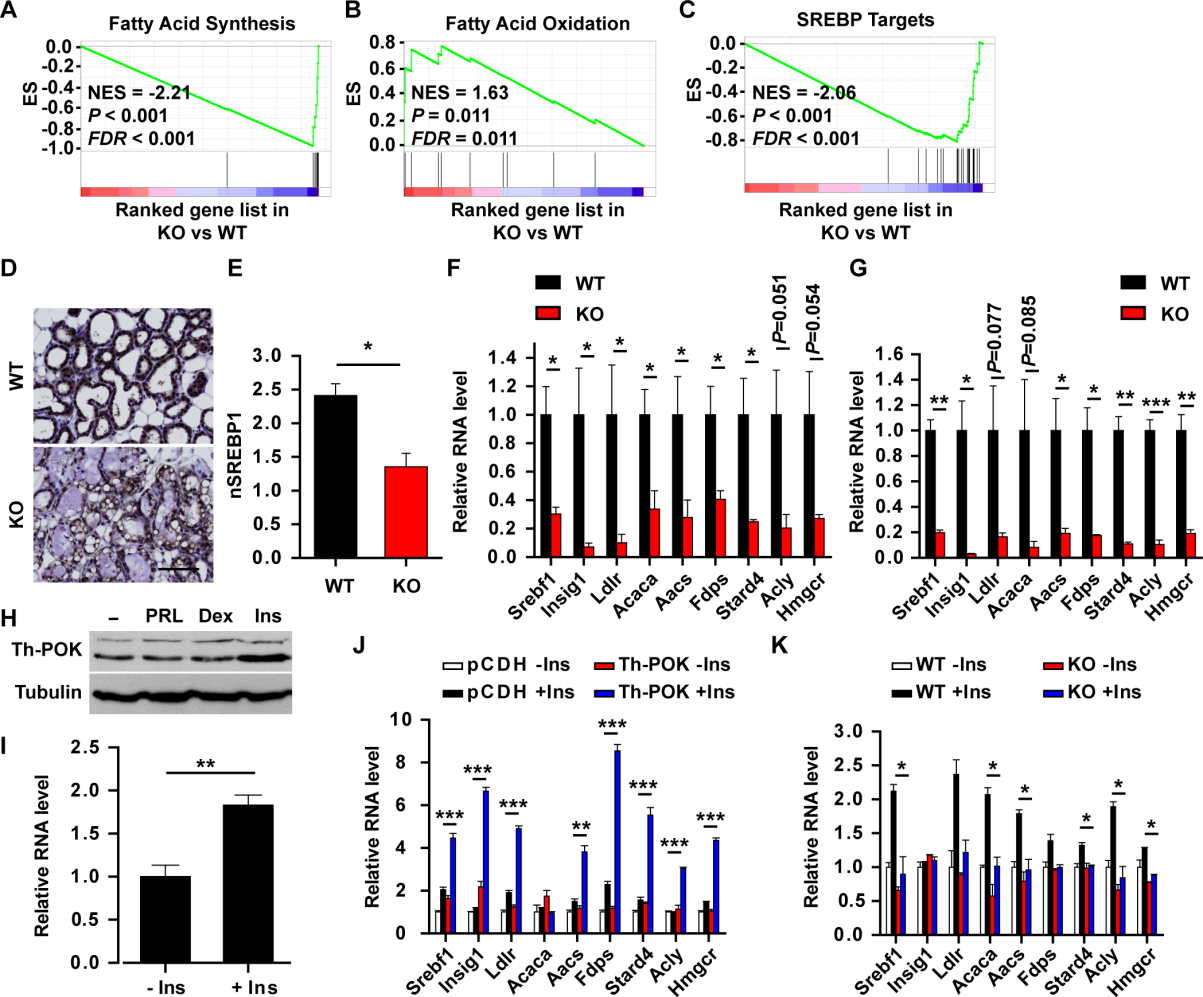
Th-POK deficiency impairs SREBP pathway in lactating mammary glands. (A-C) GSEA analyses of genes regulating fatty acid synthesis (A) and oxidation (B) and SREBP target genes (C) in KO mammary gland compared to the WT mammary gland at lactation day 1. ES: Enrichment score. NES: normalized enrichment score. *FDR*: false discovery rate. (D) Immunostaining of SREBP1 on mammary gland sections from WT and KO mice at lactation day 1. Scale bar: 100μm. (E) Score of nuclear SREBP1 in WT or KO mammary epithelial cells at lactation day 1 (N = 3, five fields/mice). (F) RT-qPCR analyses of SREBP target gene expression in mammary glands (N = 4 mammary glands from individual mice) at lactation day 2. (G) RT-qPCR analyses of SREBP target gene expression in isolated mammary epithelial cells (N = 3) from WT and KO mice at lactation day 2. (H) Western blot analysis of Th-POK expression in HC11 mammary epithelial cells treated with prolactin, dexamethasone or insulin. (I) RT-qPCR analyses of Th-POK expression in HC11 cells treated with or without insulin (N = 3). (J) RT-qPCR analyses of SREBP target gene expression in Th-POK-expressing HC11 cells treated with or without insulin (N = 3). (K) RT-qPCR analyses of SREBP target gene expression in primary WT and KO mammary epithelial cells treated with or without insulin (N = 2). Data are presented as mean ± SEM. *P < 0.05, **P < 0.01, ***P < 0.001.

Expression of the genes regulating lipid biosynthesis increases at the onset of lactation [17, 27, 34]. SREBPs are the key transcription factors regulating fatty acid and cholesterol biosynthesis [31–33]. SREBP-1 was proposed as a critical regulator of milk lipid synthesis and secretion at lactation [17, 27, 34]. Genes regulating fatty acid synthesis (S5A and S5D Fig) [27] and SREBP target genes (S5C and S5F Fig) [46] were enriched, whereas genes regulating fatty acid oxidation (S5B and S5E Fig) [27] were negatively enriched in the lactating mammary glands compared to that at late pregnancy. The SREBP signature correlated to Th-POK expression levels during pregnancy and lactation (S5G and S5H Fig). Consistent to the defects in milk lipid production in the KO mammary glands, the SREBP signature and genes regulating fatty acid synthesis were enriched in the lactating WT mammary glands, whereas genes regulating fatty acid oxidation were enriched in the lactating KO mammary glands (Fig 5A–5C). Higher levels of nuclear SREBP1 were detected in the lactating wild-type mammary glands, compared to the KO mammary glands (Fig 5D and 5E). RT-qPCR analyses revealed that genes in the SREBP pathway were significantly downregulated in the lactating KO mammary glands and isolated mammary epithelial cells (Fig 5F and 5G). Th-POK is upregulated at late pregnancy and lactation (Fig 1K–1M and S1 Fig). Lactogenic factors insulin, but not prolactin or dexamethasone, induced Th-POK expression in HC11 mammary epithelial cells (Fig 5H and 5I). Expression of Th-POK in HC11 cells potentiated SREBP target gene expression (Fig 5J), whereas Th-POK deficiency compromised SREBP target gene expression in primary mammary epithelial cells upon insulin stimulation (Fig 5K), suggesting Th-POK regulates insulin-induced SREBP pathway by cell autonomous mechanism in mammary epithelial cells.

### Th-POK regulates SREBP through IRS-1-Akt-mTOR pathway

**Fig 6:**
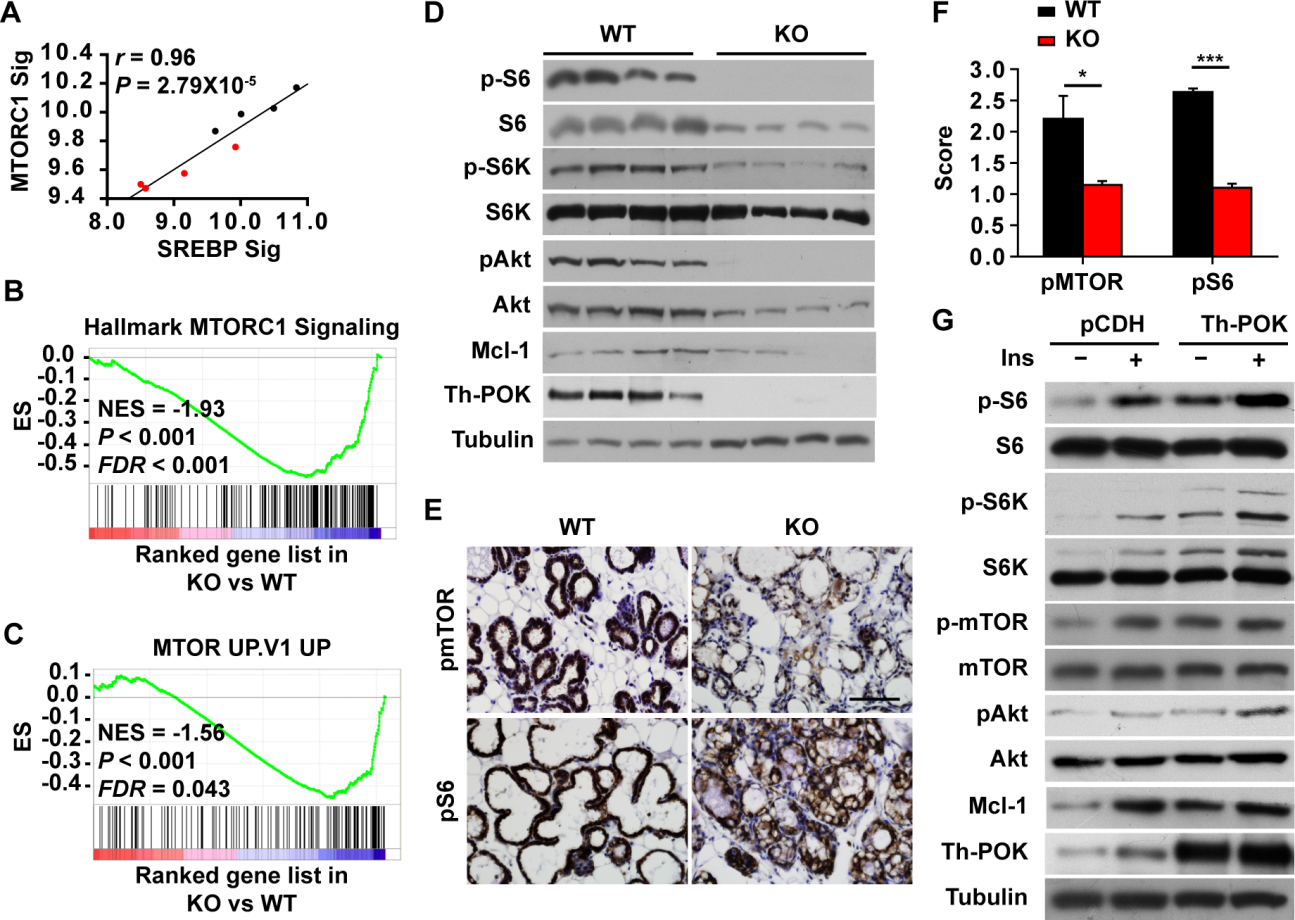
Th-POK regulates insulin-induced activation of mTOR pathway. (A) SREBP signature is significantly correlated to mTORC1 signature in lactating mammary glands. Black dots: lactating wild-type mammary glands; red dots: lactating knockout mammary glands. (B and C) GSEA analyses of mTORC1 gene signature (B) and genes upregulated by mTOR (C) in KO mammary gland compared to the WT mammary gland at lactation day 1. ES: Enrichment score. NES: normalized enrichment score. *FDR*: false discovery rate. (D) Western blot analyses of Akt-mTOR pathway in mammary glands (4 biological replicates) from WT and KO mice at lactation day 2. (E) Immunostaining of pmTOR and pS6 on mammary gland sections from WT and KO mice at lactation day 1. Scale bar: 100μm. (F) Score of pmTOR and pS6 in WT or KO mammary epithelial cells at lactation day 1 (N = 3, five fields/mice). Data are presented as mean ± SEM. *P < 0.05, ***P < 0.001. (G) Western blot analyses of Akt-mTOR pathway in Th-POK-expressing HC11 cells treated with insulin.

Akt-Mammalian target of rapamycin (mTOR) signaling is a central regulator of growth and metabolism [19, 37, 47–50]. SREBP and MTORC1 signatures correlated to each other during pregnancy and lactation (S5I and S5J Fig), and in the WT and KO mammary glands at lactation day 1 (Fig 6A), supporting previous findings that mTOR promotes lipid synthesis by activating SREBP-1 [19, 35–37, 50–53]. MTORC1 signature [41] and genes upregulated by mTOR signaling [54] were significantly enriched in the lactating mammary glands (S6A–S6D Fig). mTOR signaling was active at late pregnancy that was further activated upon parturition (S6E Fig). These promoted us to study if Th-POK functions through mTOR in the mammary glands. The MTORC1 signature [41] correlated to Th-POK expression levels during pregnancy and lactation (S6F and S6G Fig), and were negatively enriched in the KO mammary glands, compared to the WT mammary glands (Fig 6B and 6C). mTOR signaling was less activated in the alveolar epithelial cells in the KO mice at late pregnancy that was minimally further activated upon parturition (S6E Fig). Phosphorylation levels of Akt, mTOR, S6 kinase and S6 were significantly decreased in the lactating KO mammary glands (Fig 6D–6F and S6E Fig). Overexpression of Th-POK in HC11 cells significantly enhanced insulin-induced activation of Akt-mTOR signaling (Fig 6G). Mcl-1 is a key pro-survival factor during lactation, which is induced by mTORC1 signaling [49]. In the lactating Th-POK KO mammary glands, Mcl-1 protein levels were reduced to ~50% of that in the WT tissues (Fig 6D). Th-POK significantly induced Mcl-1 expression in HC11 cells (Fig 6G).

**Fig 7:**
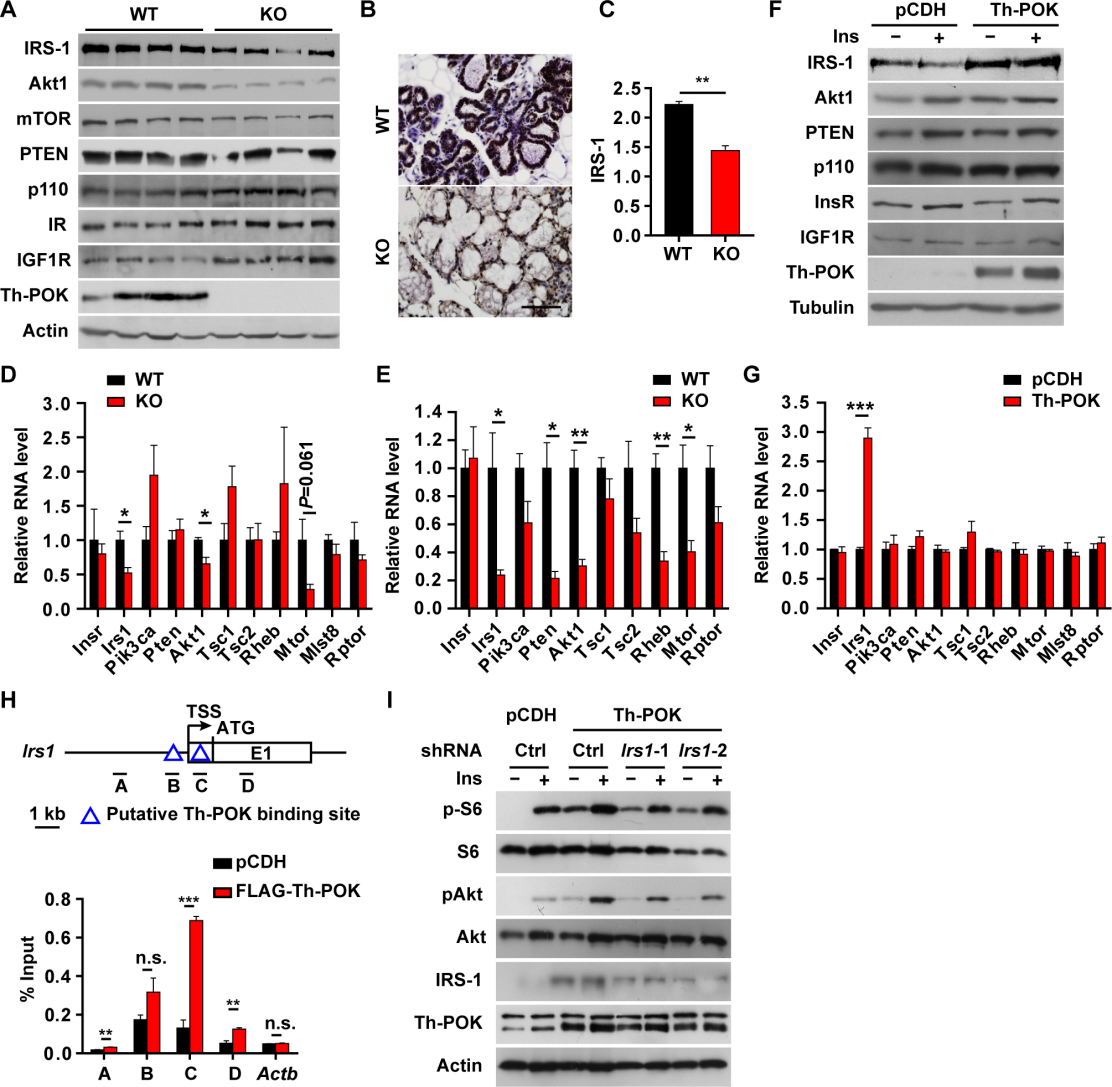
Th-POK regulates mTOR pathway through IRS-1. (A) Western blot analyses of Akt-mTOR pathway in mammary glands (4 biological replicates) from WT and KO mice at lactation day 2. (B) Immunostaining of IRS-1 on mammary gland sections from WT and KO mice at lactation day 1. Scale bar: 100μm. (C) Score of IRS-1 in WT or KO mammary epithelial cells at lactation day 1 (N = 3, five fields/mice). (D) RT-qPCR analyses of genes in Akt-mTOR pathway in mammary glands (N = 4) at lactation day 2. (E) RT-qPCR analyses of genes in Akt-mTOR pathway in isolated mammary epithelial cells (E, N = 3) from WT and KO mice at lactation day 2. (F) Western blot analyses of Akt-mTOR pathway in Th-POK-expressing HC11 cells treated with insulin. (G) RT-qPCR analyses of genes in Akt-mTOR pathway in HC11 cells expressing Th-POK (N = 3). (H) Schematic diagram of predicted Th-POK binding sites and regions amplified with corresponding PCR primers to detect ChIP products in *Irs1* locus (left). Lysates from FLAG-tagged Th-POK-expressing HC11 cells were prepared for the ChIP assay with anti-FLAG antibody (N = 3) (right). HC11 cells transduced with empty vector was used as control. *Actb* (β-actin) was used as negative control. (I) Western blot of analyses of Akt-mTOR pathway in Th-POK-expressing, IRS-1 knock-down HC11 cells. Data are presented as mean ± SEM. *P < 0.05, **P < 0.01, ***P < 0.001. n.s.: not significant.

Th-POK deficiency downregulated insulin receptor substrate-1 (IRS-1), Akt1 and mTOR expression in lactating mammary glands (Fig 7A–7E). Overexpression of Th-POK in HC11 cells significantly upregulated IRS-1 expression (Fig 7F and 7G). IRS-1 is the major adaptor protein transducing insulin signaling. Mammary IRS-1 expression levels were increased upon parturition [55], and were significantly correlated to that of Th-POK and insulin receptor during pregnancy and lactation (S7 Fig). IRS-1-deficient mice displayed a reduction in insulin-dependent Akt activation in the mammary glands and reduced lactation capacity [55]. Potential Th-POK-binding site in the *Irs1* 5'-UTR, but not in the proximal promoter, effectively bound Th-POK (Fig 7H), suggesting Th-POK directly regulated IRS-1 expression. IRS-1 knockdown in HC11 cells significantly decreased Akt and S6 phosphorylation elicited by Th-POK overexpression (Fig 7I).

## Discussion

Transition from pregnancy to lactation upon parturition is a critical event in producing sufficient milk and nutrients to the infants [12, 14, 17]. Such a process, however, is not well understood. In this report, we show that Th-POK, a key determinant in T cell development, is an important regulator of mammary gland lactation onset upon parturition. Th-POK is restrictedly expressed in the luminal epithelial cells in mammary glands that activates mTOR-SREBP signaling and lipid biosynthesis at the transition from pregnancy to lactation.

Th-POK is expressed in the mammary glands in a manner similar to GATA-3 (Fig 1), a transcription factor most highly expressed in the mammary glands [15, 16]. GATA-3-deficient mice exhibited severe defects in mammary gland development during puberty and defective alveolar differentiation during pregnancy [15, 16]. Although Th-POK is the master regulator in T cell fate determination downstream of GATA-3 [6, 7], Th-POK is not involved in regulating mammary epithelial cell fate determination (S2 Fig). Th-POK-deficient mice were largely normal in mammary gland development during puberty and pregnancy. GATA-3 regulates T cell differentiation via both Th-POK-dependent and -independent mechanisms [6]. GATA-3 may regulate mammary luminal cell differentiation independent of Th-POK. Effectors, e.g. FOXA1 [15] but not Th-POK, may account for the luminal cell fate determination in the mammary glands.

Th-POK expression is upregulated at late pregnancy and is maintained at high levels at lactation (Fig 1). Such expression kinetics indicates that Th-POK may function primarily at the lactation stage. Expression of milk proteins, e.g. caseins and WAP, is turned on at early- to mid-pregnancy [17]. In contrast, milk lipid synthesis mainly occurs at late pregnancy, and is sharply upregulated upon parturition [17]. The upregulated milk protein expression and secretion of lipid into the lumen are indicative of successful functional differentiation [12] and lactation onset [17], respectively. Deficiency of Th-POK did not affect milk protein production, but substantially decreased the amount of milk lipid (Fig 2). Such phenotypic abnormality further indicates that Th-POK is not involved in the regulation of mammary gland differentiation, but responsible for regulating efficient transition from pregnancy to lactation.

Interaction between XOR and BTN and XOR oxidoreductase activity are essential to the secretion of CLDs [21, 23, 26]. Th-POK, however, did not regulate XOR expression or activity. Rather, Th-POK regulates mTOR-SREBP pathway. Mammary epithelial cells coordinately upregulate multiple biosynthetic pathways in order to fulfill vast demand for milk lipid and protein production during lactation. Akt-mTOR signaling is a central regulator of metabolism [19, 37, 47–49]. Blockade of mTOR signaling results in lactational insufficiency and decrease in pup weight [49]. Akt-mTOR signaling promotes lipid synthesis by activating SREBP-1 [19, 35–37, 50–53], the latter was proposed as a critical regulator of transition from pregnancy to lactation [17, 20, 27, 34]. mTOR and SREBP-1 pathways were coordinately activated in mammary glands upon parturition. Th-POK regulates insulin-induced mTOR signaling and SREBP-1 activity by binding to *Irs1* locus and regulating IRS-1 expression (Fig 7). Deficiency of Th-POK compromised Akt-mTOR signaling and downstream SREBP activity. Conversely, insulin induced Th-POK expression in mammary epithelial cells (Fig 5). Therefore, Th-POK may function as a feed forward regulator of insulin signaling in the mammary glands. Interestingly, cytosolic NEFA level was increased in Th-POK deficient mammary epithelial cells (Fig 2). Expression of genes responsible for TAG synthesis was substantially decreased in Th-POK deficient mammary epithelial cells (Fig 2). mTOR signaling not only promotes lipid biogenesis, but also inhibits lipolysis [56]. Lipa expression was upregualted in Th-POK deficient mammary epithelial cells (Fig 2). High NEFA levels in Th-POK deficient mammary epithelial cells may be due to impaired TAG synthesis and enhanced TAG breakdown, as a result of reduced mTOR signaling. In addition to reduced mTORC1-mediated SREBP activation, high level cytosolic NEFA may further exert feedback inhibition on SREBP.

Besides IRS-1, expression of multiple components in the insulin-Akt-mTOR pathway were altered in the Th-POK deficient lactating mammary glands, e.g. Akt1 and mTOR. Akt1 were more significantly downregulated in the Th-POK deficient lactating mammary glands at the protein level than the mRNA level (Fig 7). Although Akt1 mRNA level was not changed, its protein level was significantly higher in the Th-POK expressing HC11 cells (Fig 7). In addition to the transcriptional regulation, Th-POK may regulate Akt-mTOR pathway at the protein level. Th-POK may also regulate the response of mammary epithelial cells to other lactogenic stimuli, e.g. prolactin, which coordinates the expression of milk protein and a subset of mammary epithelial cell specific lipogenic gene expression [30].

Defective transition from pregnancy to lactation in the Th-POK-deficient lactating mice is mainly attributed to the defects in the mammary epithelial cells in an autonomous manner. Transplanted Th-POK-deficient, but not the wild-type, mammary epithelial cells show defective transition upon parturition, independent of the Th-POK expression status in the mammary gland microenvironment (Fig 4). The possibility exists that Th-POK in the microenvironment may as well affect milk lipid biosynthesis and secretion in the alveolar epithelial cells. Although the difference was not statistically significant, size of the CLDs in the Th-POK deficient recipient mice was slightly larger than the ones in the wild-type recipient mice (Fig 4). Th-POK deficiency impairs CD4 T cell differentiation [1–3]. Infiltration of lymphocytes into the mammary gland during lactation is critical for the passive immunity to the newborn [57, 58]. It warrants further investigation whether Th-POK in the infiltrated immune cells regulates milk secretion process. Th-POK is recently reported to be expressed in the brown adipose and regulates brown fat development [59], providing another line of evidence that Th-POK regulates metabolic processes. Th-POK deficient mice lose mammary adipose tissues at lactation, despite mammary adipose tissues are largely normal at puberty and pregnancy (Fig 2 and S2 Fig). Fatty acids for milk lipid synthesis originate either from de novo lipogenic pathways within the alveolar epithelial cells, or from dietary fat and adipose tissues [60, 61]. De nove lipogenic pathways are the major source of fatty acids for milk triacylglycerol synthesis for mice on chow diet [60–62]. Th-POK deficient dams may mobilize more fatty acids from the mammary adipose tissues, due to misregulated lipogenesis in the Th-POK deficient alveolar epithelial cells.

Premature involution is frequently observed in mouse models with defects in secretory activation/initiation, including in the Th-POK deficient mice [20, 21, 23, 25]. Defective secretory activation/initiation triggers alveolar cell apoptosis and premature involution. Alternatively, existing premature involution program may impair milk production and secretion. Although apoptosis was detected in a small population of Th-POK deficient alveolar epithelial cells at lactation, no apoptotic cell was evident at late pregnancy (Fig 3). Expression of *Cebpd* and *Socs3* was upregulated in the lactating Th-POK deficient mammary glands (Fig 3). However, Th-POK does not affect the expression of *Cebpd* and *Socs3* in HC11 mammary epithelial cells (Fig 3), indicating that Th-POK does not proactively regulate *Cebpd* and *Socs3* expression and the premature involution program. mTOR pathway activation was impaired in the Th-POK deficient mammary glands. The difference in mTOR activity is already evident at late pregnancy (S6 Fig). Unlike in the wild-type mammary glands, mTOR signaling is not further activated in the Th-POK deficient mammary glands upon parturition (S6 Fig). Therefore, it is more likely that mTOR signaling is compromised in the Th-POK deficient alveolar epithelial cells and the resultant inability to initiate adequate milk secretion triggers involution program, i.e. Cebpd and Socs3 expression and alveolar epithelial cell apoptosis. Mcl-1 was recently reported as a key pro-survival factor during lactation [49]. Th-POK induces Mcl-1 expression (Fig 6). It warrants further investigation whether lower level Mcl-1, as a result of impaired mTOR signaling, is responsible for the induction of the premature involution in the lactating Th-POK KO mammary glands.

In summary, we discovered a novel physiological function of Th-POK in mammary glands. Th-POK is expressed in the mammary luminal epithelial cells. Unlike its role in T cell fate determination, Th-POK is not required for mammary epithelial cell fate determination and mammary gland development during puberty and pregnancy. Upregulated Th-POK expression in mammary glands at late pregnancy and lactation is necessary for mTOR-SREBP pathway-mediated lipid synthesis in the mammary epithelial cells. Ablation of Th-POK impairs secretory activation and efficient milk production upon parturition. These data demonstrate Th-POK as a critical regulator of mammary gland functions in lactation. Lineage-restrictedly expressed Th-POK exerts distinct and independent biological functions in T cells and mammary epithelial cells in a tissue-specific manner.

## Materials and methods

### Ethics statement

The mice were housed in a specific pathogen-free environment at the Shanghai Institute of Biochemistry and Cell Biology (SIBCB) and treated in strict accordance with protocols approved by the Institutional Animal Care and Use Committee of Shanghai Institute of Biochemistry and Cell Biology (Approval number: SIBCB-NAF-15-003-S325-006).

### Mice

Th-POK knockout mice in C57/Bl background were gift from Dr. R. Bosselut (National Institutes of Health, Bethesda, MD). The mice were fed with standard rodent chow diet. Age matched female mice were used.

### Survival rate and weight gain

Female mice were mated with males between age 10–12 weeks. Pup survival was calculated as the percentage of surviving pups in each litter at 48 hours postpartum [20]. For cross feeding experiment, 7 heterozygote pups born to wild-type female mice were fostered to each WT or KO mother at the time of parturition. The pups were weighed daily for 8 days and average pup weight was calculated. Survival of the pups was recorded to perform the Kaplan-Meier survival analysis.

### Milk analyses

Pups were removed for 3 hours at day 2 of lactation and dams were injected with 10 units of oxytocin to induce milk letdown. Milk was manually removed from the fourth mammary glands. For MFG detection, milk was diluted in PBS and stained with Bodipy @493/503 (Life Technologies). The captured images were analyzed by ImagePro Plus (v6.0, Media Cybernetics). Triacylglycerol analyses were performed following manufacturer's protocols (Triglycerides kit, Shanghai KEHUA bio-engineering CO.).

### Whole mount preparation and morphologic analysis

The carmine alum-stained whole mounts were prepared as described [63]. To measure ductal growth, the whole mounts of mammary glands stained with Carmine alum were imaged with a microscope. The captured images were imported to ImagePro Plus (v6.0, Media Cybernetics) and analyzed as described [64].

### H&E, immunofluorescent and immunohistochemical staining

Mouse mammary glands were isolated and fixed in 4% PFA followed by embedding in paraffin. Paraffin-embedded tissues were sectioned and stained with hematoxylin and eosin (H&E). The immunofluorescent and immunohistochemical staining were performed as previously described [65]. Sections were incubated at 4°C overnight with primary antibodies. Antibodies used are listed in S1 Table. H-score was used for semi-quantitative analysis of the immunohistochemical staining. Mammary epithelial cells (three mice and five randomly selected fields of each sample) were counted, and the H-score was calculated by adding the percentage of strongly stained (×3), moderately stained (×2), and weakly stained cells (×1) as described [66, 67]. H-score has a possible range of 0–3. Fore regions of the mammary glands were compared in the histological and immunohistochemical analyses.

### BrdU incorporation

Two hours before sacrifice, mice were injected intraperitoneally with 5 μl BrdU/g of body weight (Roche). Mammary glands were prepared as described above. For detection of incorporated BrdU, 7-μm sections were processed according to the manufacturer’s instructions (Roche). The BrdU-positive and total mammary epithelial cells were counted. Three mice and five randomly selected fields of each sample were analyzed.

### Mammary fat pad transplantation and analysis

Mammary fat pad transplantation experiment was performed as described [68]. Mammary epithelial cells isolated from wild-type or Th-POK-deficient mice were injected into the cleared fat pads of 3-week-old wild-type or Th-POK-deficient female mice. 8–10 weeks after surgery, the mice were mated with wild-type male mice. Reconstituted mammary glands were harvested after parturition at lactation day 2, and subjected to paraffin embedding, histology and immuno-staining.

### Primary mammary epithelial cell isolation

Mammary glands from 8- to 12-wk-old virgin female mice or mice at lactation day 2 were isolated. The minced tissue was digested in RPMI 1640 with 25 mM HEPES, 5% FBS, 1% PSQ, 300U/mL Collagenase III (Worthington) for 2 hours at 37°C. After lysis of the red blood cells, single cell suspension was obtained by sequential incubation with 0.25% Trypsin-EDTA for 5 min and 0.1 mg/mL DNase I (Sigma) for 5 min at 37°C with gentle pipetting, followed by filtration through 40-μm cell strainers. The antibodies used for flow cytometry were: PE-conjugated CD31, CD45, TER119 (BD PharMingen), CD24-PE/cy7 and CD29-APC (Biolegend). All sorting was performed using FACSAria or FCASJazz (Becton Dickinson). The purity of sorted population was routinely checked and ensured to be >95%. Cells were harvested for quantitative RT-PCR experiments. For insulin treatment experiment, primary mammary epithelial cells were seeded in DMEM (Invitrogen) supplemented with 10% FBS. Adhered cells were treated with 10μg/mL insulin for 24 hours and were harvested for quantitative RT-PCR experiments. Cytosolic triacylglycerol and nonesterified fatty acid in primary mammary epithelial cells were quantified following manufacturer's protocols (Triglycerides kit, Shanghai KEHUA bio-engineering CO., and LabAssay NEFA kit, Wako).

### Cell lines

HC11 cells (generously provided by Dr. Peng Li, Tsinghua University) were cultured in 1640 (Invitrogen) supplemented with 10% fetal bovine serum (FBS, Biochrom), 100 units/ml penicillin, 100 μg/ml streptomycin (Invitrogen), 5 μg/ml insulin (Sigma) and 20 ng/ml EGF (Abcam) in 5% CO_2_ at 37°C. The cell lines were routinely tested for mycoplasma contamination. For insulin treatment experiments, HC11 cells were cultured in the absence of insulin and EGF overnight before 5μg/mL insulin treatment for 24 hours. Cells were harvested for western blotting and quantitative RT-PCR experiments.

### Plasmids

FLAG-tagged Th-POK was subcloned into pCDH-puro lentiviral vector [11]. The shRNA sequences were cloned into the pLKO.1-puro lentiviral vector. The shRNA target sequences were 5’-GCCTGGAGTATTATGAGAACG-3’ and 5’-GCGATTTCCGAAGTTCCTTCC-3’ (mouse IRS-1). Virus packaging and infection were performed as described [65].

### Western blot analysis

Western blot analysis was performed as previously described [65]. Antibodies used are listed in S1 Table.

### Quantitative RT-PCR

Total RNA was prepared from mouse tissues or HC11 cells using Trizol reagents (Invitrogen). Equal amounts of RNA from mammary gland were subjected to quantitative RT-PCR using SYBR green with the BIO-RAD Q-PCR Systems according to the manufacturer's protocol. Relative expression levels were calculated using the comparative CT method. Gene expression levels were normalized to Actin. The primers used are listed in S2 Table.

### ChIP analysis

Real-time PCR-based ChIP analysis was performed as described previously [69]. Cells were incubated with medium containing 0.9% formaldehyde for 10 min at room temperature. Sonicated chromatin fragments averaged ~300 to 500 bp. Soluble chromatin was incubated with the M2 Anti-FLAG Agarose (Sigma). DNA was quantitated by real-time PCR. The amounts of products were determined relative to input chromatin. The primers used are listed in S2 Table.

### Microarray and GSEA analysis

Gene expression profiles of mammary gland tissues from wild-type and Th-POK knockout mice on lactation day 1 were analyzed using Agilent Mouse 4 × 44 K Gene Expression Arrays, following the manufacturer's instructions. Four independent sets of biological replicates of mammary gland samples were used. Data were normalized by Quantile algorithm, Gene Spring Software 11.0 (Agilent technologies, USA). Gene expression data are available at the Gene Expression Omnibus (GEO) under accession number GSE97566. Gene expression data of mammary glands during pregnancy, lactation, and involution were downloaded from GEO (GDS2843 and GDS1805) [17, 27]. Gene set enrichment analysis (GSEA) were performed on gene signatures obtained from the MSigDB database v5.0 (March 2015 release) [27, 41, 46, 54]. Statistical significance was assessed by comparing the enrichment score to enrichment results generated from 1,000 random permutations of the gene set to obtain *P* values (nominal *P* value) and false discovery rate (*FDR*).

### Statistics

Sample size for each figure is denoted in the figure legends. Statistical significance between conditions was assessed by two-tailed Student’s *t*-tests. For multiple group comparison, two-way Anova analysis was performed followed by Bonferroni's multiple comparison test. All error bars represent SEM, and significance is denoted as *P < 0.05, **P < 0.01 and ***P < 0.001. n.s. denotes not significant.

## Supporting information

S1 Fig Th-POK expression increases at late pregnancy and lactation. Th-POK expression at pregnancy and lactation in microarray datasets GDS2843 (A) and GDS1805 (B). Data are presented as mean ± SEM. *P < 0.05, **P < 0.01, ***P < 0.001, compared to the first time point.
(TIF)

S2 Fig Normal mammary gland development during puberty and pregnancy in Th-POK knockout mice. (A) Carmine-stained whole-mounted mammary glands from 5-, 7- and 10-week-old WT and KO virgin mice. Scale bar: 4mm. (B) Average ductal length of the fourth inguinal mammary glands (WT = 5, 8, 5; KO = 3, 7, 4 for 5-, 7-, and 10-week-old mice). (C) Numbers of terminal end buds in the fourth inguinal mammary glands from 5-week-old virgin mice (WT = 6, KO = 4). (D) Hematoxylin-and-eosin-stained sections of mammary glands from 5-, 7- and 10-week-old WT and KO mice. Scale bars: 100μm. (E) Carmine-stained whole-mounted mammary glands from WT and KO mice at pregnancy day 5.5 or 12.5. Scale bar: 2mm. (F) Hematoxylin-and-eosin-stained sections of mammary glands from WT and KO mice at pregnancy day 5.5 or 12.5. Scale bars: 100μm. (G) BrdU analysis of mammary glands from WT and KO mice at pregnancy day 5.5, 12.5 or 17.5. Scale bar: 25μm. (H) Quantitative analysis of BrdU analysis in (G) (N = 3, six fields/mice). Data are presented as mean ± SEM. n.s.: not significant.
(TIF)

S3 Fig Normal milk protein production in Th-POK knockout mice. (A) RT-qPCR analyses of expression of β-casein, whey acidic protein (WAP) and α-lactalbumin in mammary glands from WT and KO mice at lactation day 2 (N = 4). Data are presented as mean ± SEM. n.s.: not significant. (B and C) Milk was collected from fourth mammary glands following oxytocin stimulation at lactation day 2. (B) Milk protein concentration was compared (N = 4 each). (C) Equal volumes of milk collected from WT or KO mice were analyzed by SDS-PAGE and coomassie brilliant blue staining.
(TIF)

S4 Fig Impaired lipid secretion in Th-POK knockout mice is not due to defects in known pathways. (A) Immunostaining of Ezrin or E-cadherin (E-Cad) on section of mammary glands from WT and KO mice at lactation day 1. Scale bar: 25μm. (B) RT-qPCR analyses of expression of perilipin2 (*Plin2*), butyrophilin (*Btn*), xanthine oxidoreductase (*Xor*) and *Cidea* in mammary glands from WT and KO mice at lactation day 1 (N = 4). (C) Western blot analysis of XOR expression and Src phosphorylation in mammary glands from WT and KO mice at lactation day 2. (D) XOR activity from WT and KO mice at lactation day 2 (N = 4). Data are presented as mean ± SEM. n.s.: not significant. (E) GSEA data showing the enrichment of Src oncogenic signature in mammary glands at lactation day 1, compared to those at pregnancy day 19 (upper panel). No significant difference between mammary glands from WT and KO mice at lactation day 1 (bottom panel). NES: normalized enrichment score. *FDR*: false discovery rate.
(TIF)

S5 Fig SREBP pathway is activated in mammary glands upon parturition. (A-F) GSEA analyses of genes regulating fatty acid synthesis (A and D), genes regulating fatty acid oxidation (B and E), and SREBP gene signature (C and F) in mammary glands at lactation day 1 compared to those at pregnancy day 19 in microarray dataset GDS2843 (A-C) or at pregnancy day 17 in microarray dataset GDS1805 (D-F). NES: normalized enrichment score. *FDR*: false discovery rate. (G and H) Correlation of Th-POK expression to SREBP signature at pregnancy and lactation in microarray datasets GDS2843 (G) and GDS1805 (H). (I and J) Correlation of SREBP signature to MTORC1 signature at pregnancy and lactation in microarray datasets GDS2843 (I) and GDS1805 (J).
(TIF)

S6 Fig mTOR pathway is activated in mammary glands upon parturition, and is coactivated with SREBP1. (A-D) GSEA analyses of mTORC1 gene signature (A and C) and genes upregulated by mTOR (B and D) in mammary glands at lactation day 1 compared to those at pregnancy day 19 in microarray dataset GDS2843 (A and B) or at pregnancy day 17 in microarray dataset GDS1805 (C and D). NES: normalized enrichment score. *FDR*: false discovery rate. (E) Immunostaining of pS6 on mammary gland sections from WT and KO mice at pregnancy day 17.5 (P17.5) and lactation day 1 (L1). Scale bar: 100μm. (F and G) Correlation of Th-POK expression to MTORC1 signature at pregnancy and lactation in microarray datasets GDS2843 (E) and GDS1805 (F).
(TIF)

S7 Fig Expression of Th-POK, insulin receptor (*Insr*) and IRS-1 (*Irs1*) are highly correlated to each other at pregnancy and lactation in microarray datasets GDS2843 and GDS1805. (TIF)

S1 Table Antibodies used in the study. (DOCX)

S2 Table Primer used for RT-qPCR. (DOCX)
