# Supplementary figures and images for: Th-POK regulates mammary gland lactation through mTOR-SREBP pathway
Source: PLoS Genet. 2018 Feb 8;14(2):e1007211. doi: 10.1371/journal.pgen.1007211 (PMC5821406; doi:10.1371/journal.pgen.1007211)

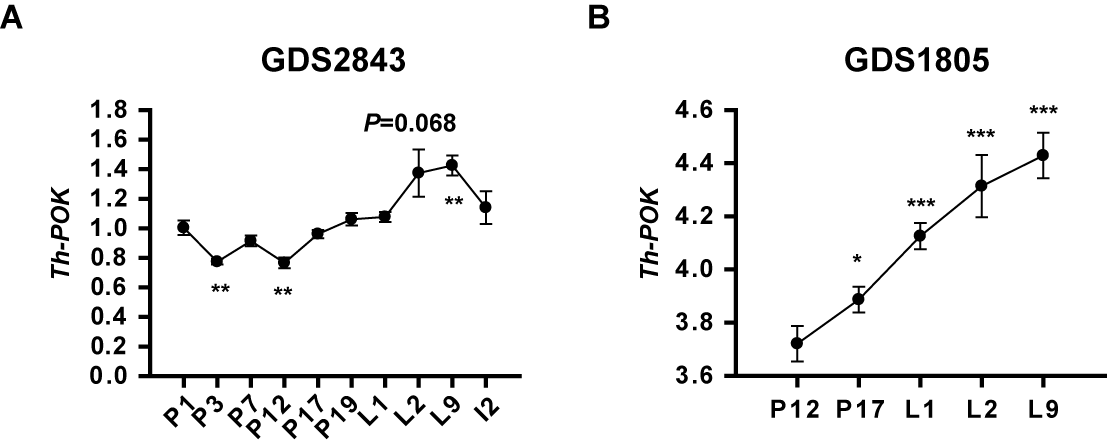

Supplement: S1 Fig — Th-POK expression at pregnancy and lactation in microarray datasets GDS2843 (A) and GDS1805 (B). Data are presented as mean ± SEM. *P < 0.05, **P < 0.01, ***P < 0.001, compared to the first time point. (TIF) [file pgen.1007211.s001.tif]

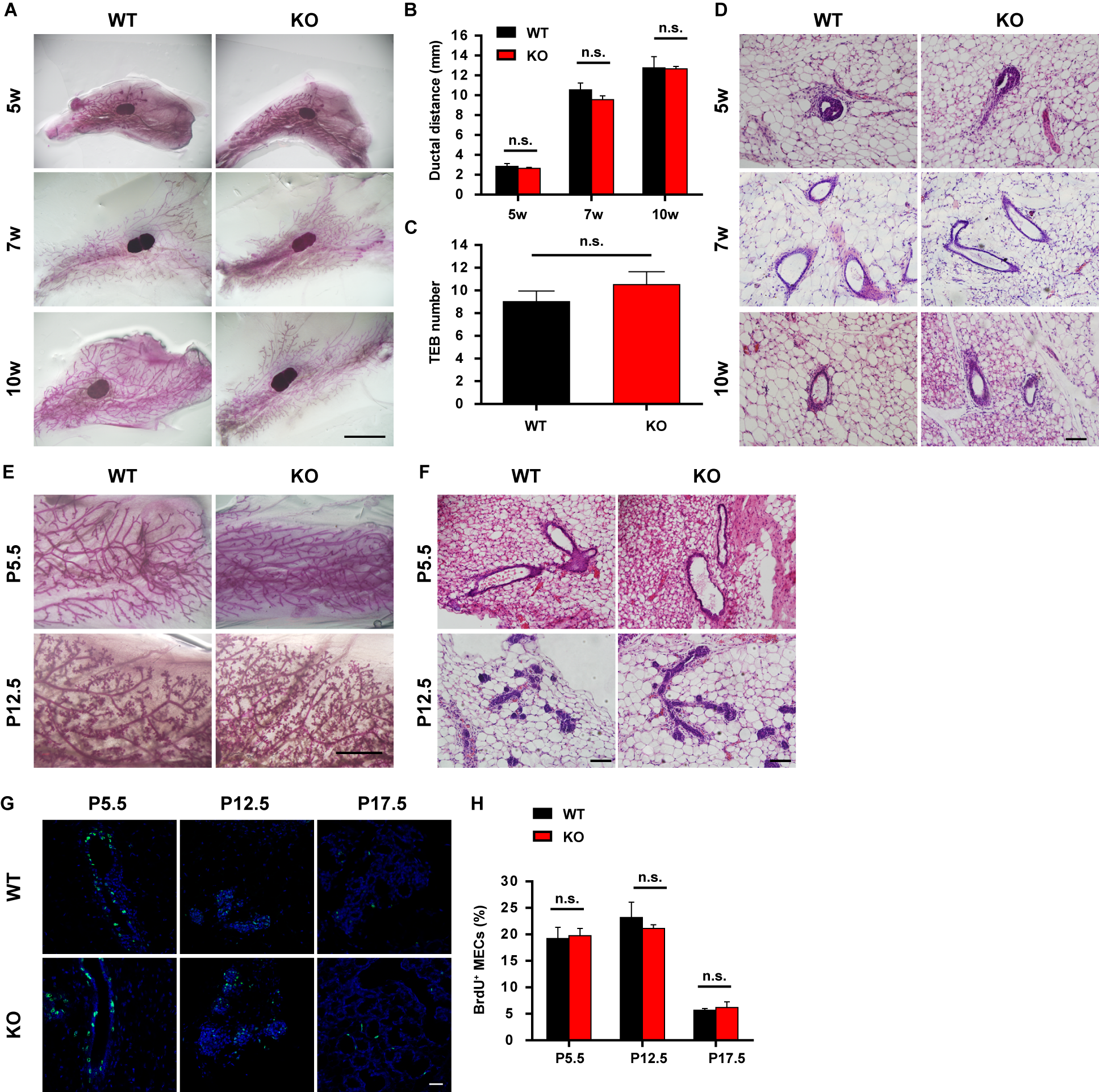

Supplement: S2 Fig — (A) Carmine-stained whole-mounted mammary glands from 5-, 7- and 10-week-old WT and KO virgin mice. Scale bar: 4mm. (B) Average ductal length of the fourth inguinal mammary glands (WT = 5, 8, 5; KO = 3, 7, 4 for 5-, 7-, and 10-week-old mice). (C) Numbers of terminal end buds in the fourth inguinal mammary glands from 5-week-old virgin mice (WT = 6, KO = 4). (D) Hematoxylin-and-eosin-stained sections of mammary glands from 5-, 7- and 10-week-old WT and KO mice. Scale bars: 100μm. (E) Carmine-stained whole-mounted mammary glands from WT and KO mice at pregnancy day 5.5 or 12.5. Scale bar: 2mm. (F) Hematoxylin-and-eosin-stained sections of mammary glands from WT and KO mice at pregnancy day 5.5 or 12.5. Scale bars: 100μm. (G) BrdU analysis of mammary glands from WT and KO mice at pregnancy day 5.5, 12.5 or 17.5. Scale bar: 25μm. (H) Quantitative analysis of BrdU analysis in (G) (N = 3, six fields/mice). Data are presented as mean ± SEM. n.s.: not significant. (TIF) [file pgen.1007211.s002.tif]

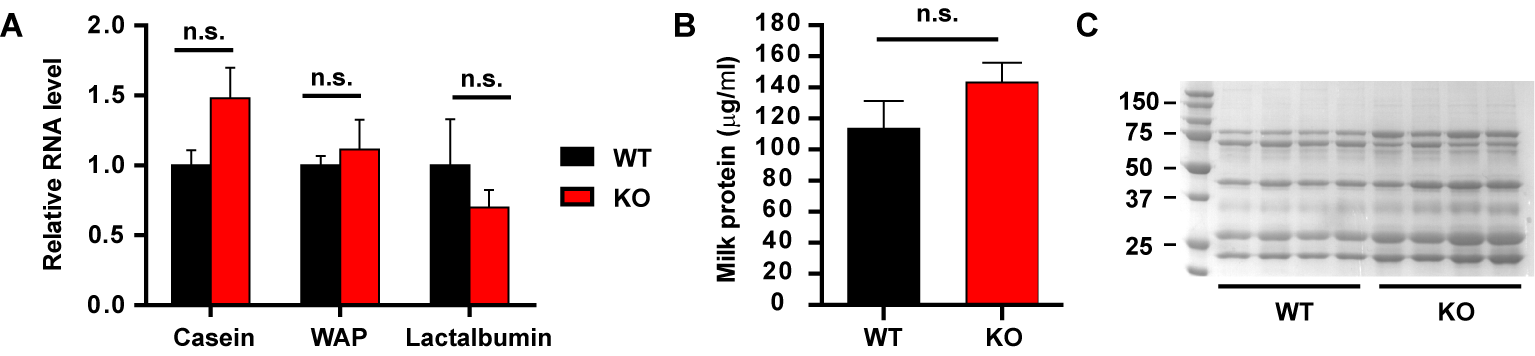

Supplement: S3 Fig — (A) RT-qPCR analyses of expression of β-casein, whey acidic protein (WAP) and α-lactalbumin in mammary glands from WT and KO mice at lactation day 2 (N = 4). Data are presented as mean ± SEM. n.s.: not significant. (B and C) Milk was collected from fourth mammary glands following oxytocin stimulation at lactation day 2. (B) Milk protein concentration was compared (N = 4 each). (C) Equal volumes of milk collected from WT or KO mice were analyzed by SDS-PAGE and coomassie brilliant blue staining. (TIF) [file pgen.1007211.s003.tif]

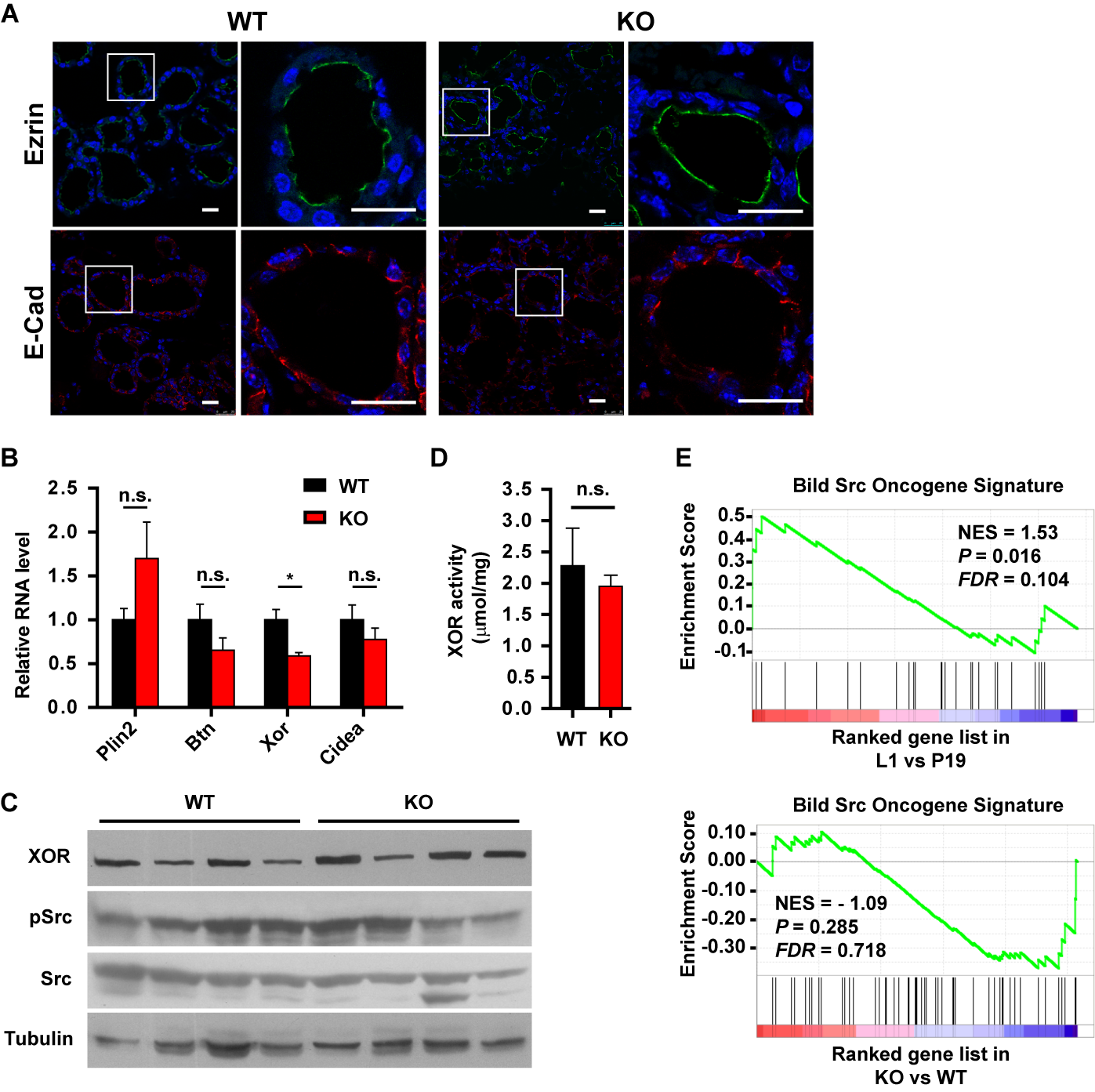

Supplement: S4 Fig — (A) Immunostaining of Ezrin or E-cadherin (E-Cad) on section of mammary glands from WT and KO mice at lactation day 1. Scale bar: 25μm. (B) RT-qPCR analyses of expression of perilipin2 (Plin2), butyrophilin (Btn), xanthine oxidoreductase (Xor) and Cidea in mammary glands from WT and KO mice at lactation day 1 (N = 4). (C) Western blot analysis of XOR expression and Src phosphorylation in mammary glands from WT and KO mice at lactation day 2. (D) XOR activity from WT and KO mice at lactation day 2 (N = 4). Data are presented as mean ± SEM. n.s.: not significant. (E) GSEA data showing the enrichment of Src oncogenic signature in mammary glands at lactation day 1, compared to those at pregnancy day 19 (upper panel). No significant difference between mammary glands from WT and KO mice at lactation day 1 (bottom panel). NES: normalized enrichment score. FDR: false discovery rate. (TIF) [file pgen.1007211.s004.tif]

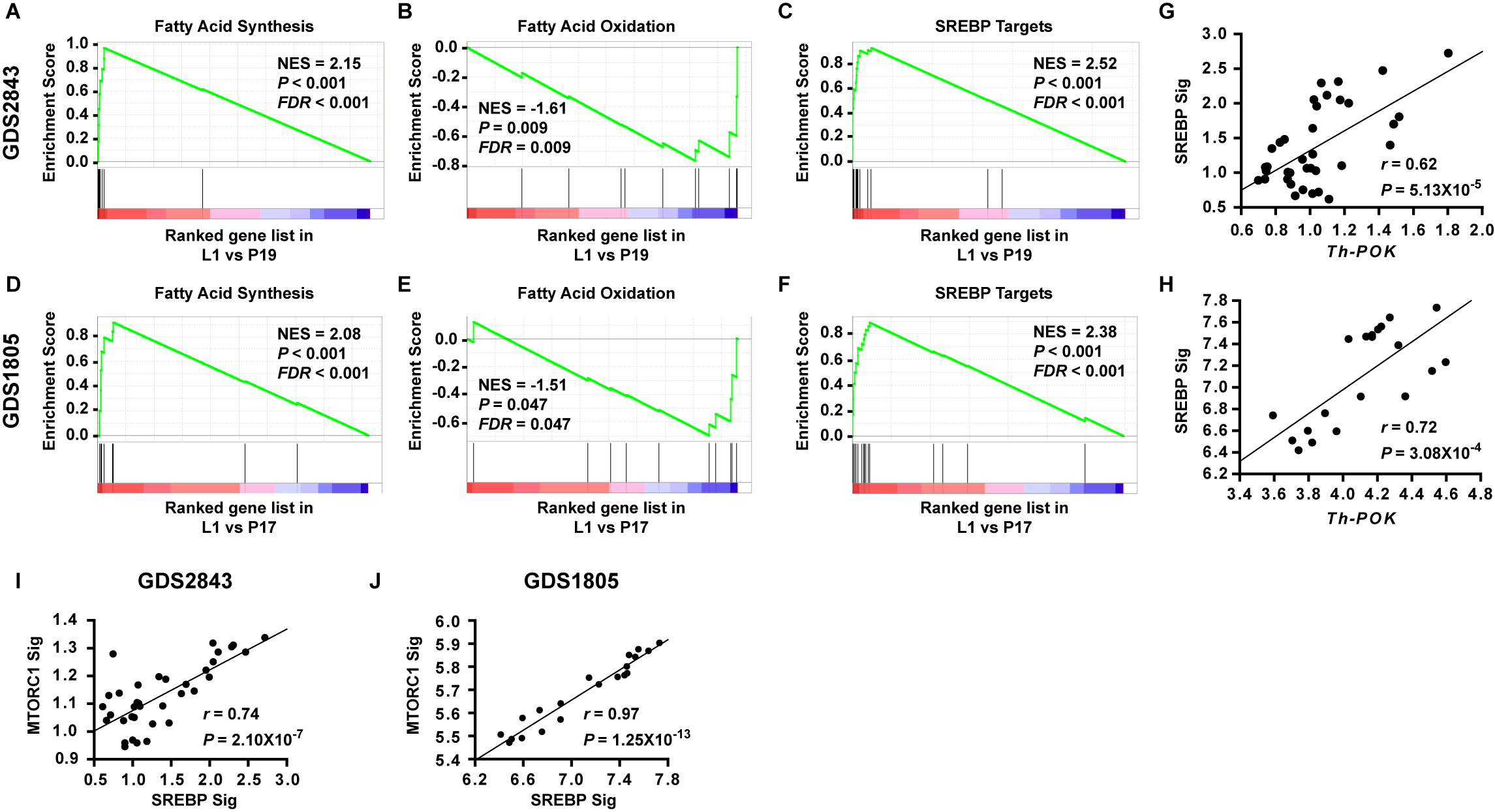

Supplement: S5 Fig — (A-F) GSEA analyses of genes regulating fatty acid synthesis (A and D), genes regulating fatty acid oxidation (B and E), and SREBP gene signature (C and F) in mammary glands at lactation day 1 compared to those at pregnancy day 19 in microarray dataset GDS2843 (A-C) or at pregnancy day 17 in microarray dataset GDS1805 (D-F). NES: normalized enrichment score. FDR: false discovery rate. (G and H) Correlation of Th-POK expression to SREBP signature at pregnancy and lactation in microarray datasets GDS2843 (G) and GDS1805 (H). (I and J) Correlation of SREBP signature to MTORC1 signature at pregnancy and lactation in microarray datasets GDS2843 (I) and GDS1805 (J). (TIF) [file pgen.1007211.s005.tif]

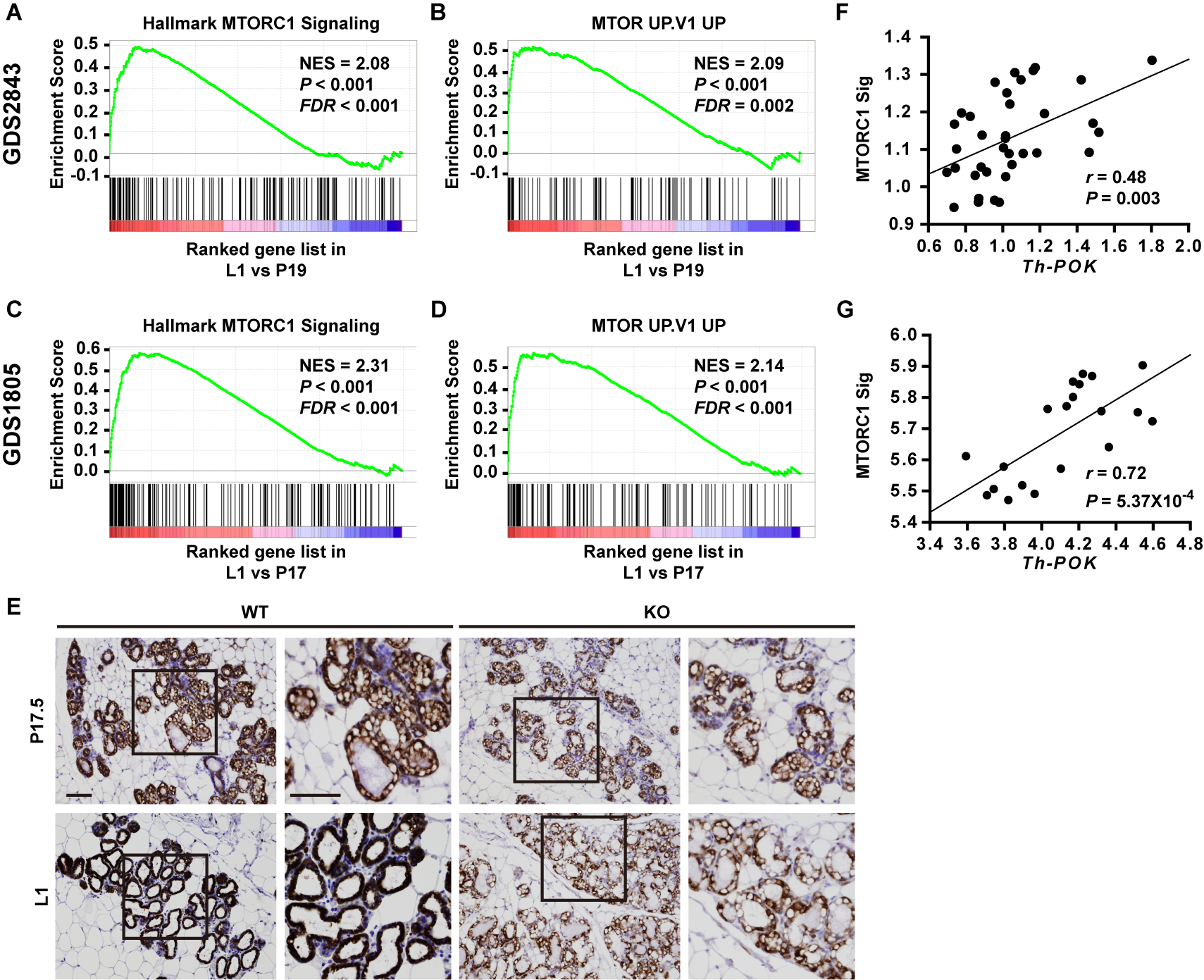

Supplement: S6 Fig — (A-D) GSEA analyses of mTORC1 gene signature (A and C) and genes upregulated by mTOR (B and D) in mammary glands at lactation day 1 compared to those at pregnancy day 19 in microarray dataset GDS2843 (A and B) or at pregnancy day 17 in microarray dataset GDS1805 (C and D). NES: normalized enrichment score. FDR: false discovery rate. (E) Immunostaining of pS6 on mammary gland sections from WT and KO mice at pregnancy day 17.5 (P17.5) and lactation day 1 (L1). Scale bar: 100μm. (F and G) Correlation of Th-POK expression to MTORC1 signature at pregnancy and lactation in microarray datasets GDS2843 (E) and GDS1805 (F). (TIF) [file pgen.1007211.s006.tif]

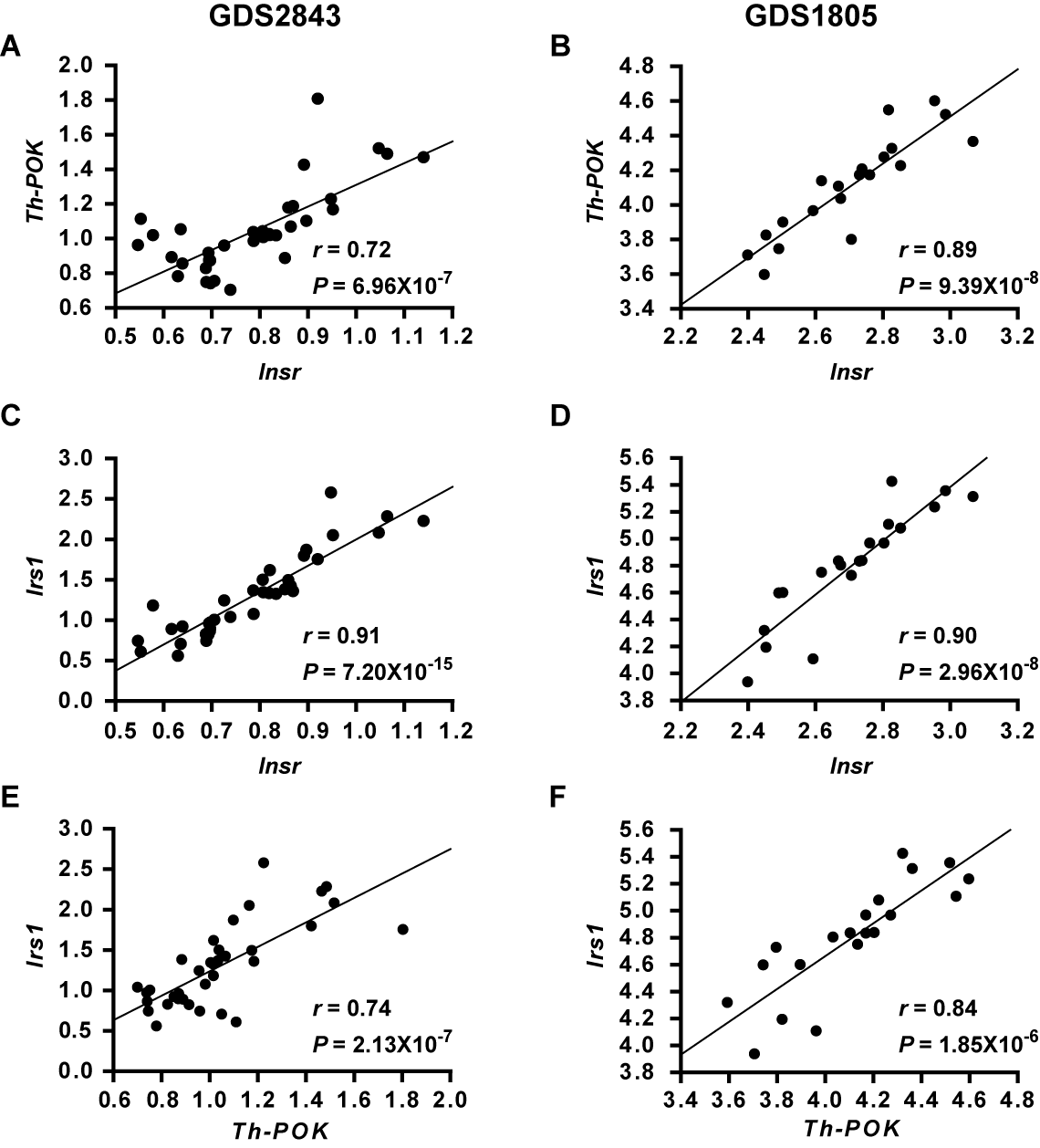

Supplement: S7 Fig — (TIF) [file pgen.1007211.s007.tif]
