# Supplementary material for: Th-POK regulates mammary gland lactation through mTOR-SREBP pathway
Source: PLoS Genet. 2018 Feb 8;14(2):e1007211. doi: 10.1371/journal.pgen.1007211 (PMC5821406; doi:10.1371/journal.pgen.1007211)
Supplement: S1 Table — (DOCX) [file pgen.1007211.s008.docx]

**Table S1. Antibodies used in the study.**

| **Antibody** | **Supplier (Cat #)** | **Application/ Concentration** |
| --- | --- | --- |
| ZBTB7B (Th-POK) | Sigma (HPA006811), rabbit polyclonal | IHC, IF: 1:100 |
| ZBTB7B (Th-POK) | ProteinTech (11341-1-AP), rabbit polyclonal | WB: 1:1000 |
| Keratin 8 | DSHB (Troma1), rat monoclonal | IF: 1:100 |
| α-SMA | Sigma (A2547), mouse monoclonal | IF: 1:3000 |
| Wheat Germ Agglutinin (WGA), Alexa Fluor® 488 Conjugate | Invitrogen (W11261) | IF: 1:1000 |
| BODIPY@493/503 | Life Technologies (D3922) | 1:100 |
| ADFP/ADRP/Plin2 | Abcam (ab52356), rabbit polyclonal | IF: 1:3000 |
| Ezrin | Cell Signaling Technology (3145), rabbit polyclonal | IF: 1:100 |
| E-cadherin (24E10) | Cell Signaling Technology (3195), rabbit monoclonal | IF: 1:1000 |
| XOR | Santa Cruz (H1611), goat polyclonal | WB: 1:500 |
| Insulin receptor | Santa Cruz (Sc-711), rabbit polyclonal | WB: 1:1000 |
| IGF-1 receptor β | Cell Signaling Technology (14534), rabbit monoclonal | WB: 1:1000 |
| IRS-1 | ProteinTech (17509-1-AP), rabbit polyclonal | IHC: 1:200; WB: 1:1000 |
| PI3 Kinase p110α | Cell Signaling Technology (4249), rabbit monoclonal | WB: 1:1000 |
| PTEN | Cell Signaling Technology (9188), rabbit monoclonal | WB: 1:1000 |
| pAkt | Cell Signaling Technology (4058), rabbit monoclonal | WB: 1:1000 |
| Akt1 | Cell Signaling Technology (2938), rabbit monoclonal | WB: 1:1000 |
| Akt | Cell Signaling Technology (4685), rabbit monoclonal | WB: 1:1000 |
| Phospho-mTOR (Ser2448) | Cell Signaling Technology (2976), rabbit monoclonal | IHC: 1:100 |
| Phospho-mTOR (Ser2448) | Cell Signaling Technology (2971), rabbit monoclonal | WB: 1:1000 |
| mTOR | Cell Signaling Technology (2983), rabbit monoclonal | WB: 1:1000 |
| Phospho-p70 S6 Kinase Thr389 | Cell Signaling Technology (9205), rabbit polyclonal | WB: 1:1000 |
| p70 S6 Kinase | Cell Signaling Technology (2708), rabbit monoclonal | WB: 1:1000 |
| Phospho-S6 Ribosomal Protein (Ser240/244) | Cell Signaling Technology (5364), rabbit monoclonal | IHC: 1:2000 |
| Phospho-S6 Ribosomal Protein (Ser240/244) | Cell Signaling Technology (2215), rabbit monoclonal | WB: 1:1000 |
| S6 | Cell Signaling Technology (2217), rabbit monoclonal | WB: 1:1000 |
| Cleaved Caspase-3 | Cell Signaling Technology (9661), rabbit polyclonal | IHC: 1:100 |
| Mcl-1 | ProteinTech (16225-1-AP), rabbit polyclonal | WB: 1:1000 |
| SREBP-1 | Santa Cruz (H160), rabbit polyclonal | IHC: 1:100 |
| α-Tubulin | Sigma (T5168), mouse monoclonal | WB: 1:15000 |
| β-actin | Sigma (A2228), mouse monoclonal | WB: 1:20000 |
| Lamin B | Abcam (ab16048), rabbit polyclonal | WB: 1:3000 |
