# Supplementary material for: Th-POK regulates mammary gland lactation through mTOR-SREBP pathway
Source: PLoS Genet. 2018 Feb 8;14(2):e1007211. doi: 10.1371/journal.pgen.1007211 (PMC5821406; doi:10.1371/journal.pgen.1007211)
Supplement: S2 Table — (DOCX) [file pgen.1007211.s009.docx]

**Table S2. Primer used for RT-qPCR.**

| Mouse Th-POK | Forward | GGCGTCCGCTTCACCAGGAAT |
| --- | --- | --- |
|  | Reverse | CCCGGTGTGCAGGTGCATGT |
| Mouse Actin | Forward | CAGCCTTCCTTCTTGGGTAT |
|  | Reverse | GGTCTTTACGGATGTCAACG |
| Mouse Krt8 | Forward | TACATCAACAACCTCCGCCG |
|  | Reverse | AGCGGCTGTTGTCCATAGC |
| Mouse Krt14 | Forward | TCCAGAGATGTGACCTCCACC |
|  | Reverse | AGGGACAATACAGGGGCTCTT |
| Mouse Srebf1 | Forward | AAAGCTTGGCCTCCCAGCAGCC |
|  | Reverse | AGTGTGGCTGCAGTACAACTGGG |
| Mouse Insigl | Forward | GGACGACAGTTAGCTATGGGTG |
|  | Reverse | AGCCCAGCCTAAATGGAACA |
| Mouse Ldlr | Forward | TTTGCAGCGGGAACATTTCG |
|  | Reverse | CTGCATGAGTCTTCTGCTGC |
| Mouse Acaca | Forward | ACACAGTTTCAGAACGGCCA |
|  | Reverse | GCCACTTTAGCGTTGGTGGT |
| Mouse Aacs | Forward | GGCACAAGGAGAACGACAGA |
|  | Reverse | GTAGATAGCCGACCACACGG |
| Mouse Fdps | Forward | AGGTCCAGGACGACTACCTT |
|  | Reverse | TCTGGGTCCTTCTGCCCATA |
| Mouse Stard4 | Forward | TCCTGTGGTGTGAGTGTTGAG |
|  | Reverse | TACGGCAGACTGAGGGATCA |
| Mouse Hmgcr | Forward | CAAGGTGGTGAGAGAGGTGT |
|  | Reverse | TGACAATGTTTGCTGCGTGG |
| Mouse Casein | Forward | TTGAACTGACTGAAACTGGA |
|  | Reverse | GTCGAATTCAAATGAATGCC |
| Mouse Wap | Forward | TGTATCATCTGCCAAACCAACGAG |
|  | Reverse | GAAGGGTCTTGCTGTATAGACTTGG |
| Mouse Lactalbumin | Forward | TCTGTGGCATCTCCTGTGACAAGT |
|  | Reverse | TGGGCTTGTAGGCTTTCCAGTAGT |
| Mouse Adrp | Forward | GATTGAATTCGCCAGGAAGA |
|  | Reverse | TGGCATGTAGTCTGGAGCTG |
| Mouse Btn | Forward | CTCTTGGTACCTCTAGATGCG |
|  | Reverse | CTGAGTGGGGTGAGGGCCCA |
| Mouse XOR | Forward | CGATGACGAGGACAACGGTA |
|  | Reverse | AGCCCCAACTTTCTTCTAAGGT |
| Mouse Cidea | Forward | TGACATTCATGGGATTGCAGAC |
|  | Reverse | GGCCAGTTGTGATGACTAAGAC |
| Mouse Igfbp5 | Forward | GGCGAGCAAACCAAGATAGAGAGAG |
|  | Reverse | GGCCGGAAGACCTTGGGGGA |
| Mouse Cebpd | Forward | GAGCAGACGCCGCCCGAATC |
|  | Reverse | CTCGCAGTCCAGTGCCCAAGC |
| Mouse Socs3 | Forward | TCGGAAGACTGTCAACGGC |
|  | Reverse | CCTCTGACCCTTTTGCTCCT |
| Mouse Insr | Forward | ATGTCCCATCAAATATTGCCAAA |
|  | Reverse | CATCCGGCTGCCTCTTTCT |
| Mouse Irs1 | Forward | AATGAGGGCAACTCCCCAAG |
|  | Reverse | TCTTCATTCTGCTGTGATGTCCA |
| Mouse Pik3ca | Forward | GTGGCCCAGATGTACTGCTT |
|  | Reverse | CACCGAACAGCAAAACTCCG |
| Mouse Pten | Forward | TCCTGCAGAAAGACTTGAAGGT |
|  | Reverse | ATGTCTCTCAGCACATAGATTGT |
| Mouse Akt1 | Forward | CGCCTGATCAAGATGACAGC |
|  | Reverse | GTGGAGCTGCCAGCCG |
| Mouse Tsc1 | Forward | TAAACAGCTGAGGGGGAGGA |
|  | Reverse | GGGCCACTACCAGACTGTTC |
| Mouse Tsc2 | Forward | TGAGAGACCCAAAAGCAGAAT |
|  | Reverse | CATCAGCCTGGGCCATAGAG |
| Mouse Rheb | Forward | TTAATGTGGTTGGGTCGGGG |
|  | Reverse | GAGGACTTTCCCACAGACCG |
| Mouse Mtor | Forward | CCCACGTGGTTAGCCAGAC |
|  | Reverse | TAGCGGATATCAGGGTCAGGA |
| Mouse Mlst8 | Forward | AGCGTGTGTTAAGTGCAGGT |
|  | Reverse | GGTCATAGCCTGCAGTTGCT |
| Mouse Raptor | Forward | CCATGTGCACAGCCCATTCT |
|  | Reverse | GGGGCTCCTGTTTTCCACA |
| Mouse Lipa | Forward | CCCACCAAGTAGGTGTAGGC |
|  | Reverse | GAGTTGCATCGGGAGTGGTC |
| Mouse Lipg | Forward | TCGGCTTTTGGAGCGTCTAT |
|  | Reverse | TTTATGATGCTCATCTCGCAGC |
| Mouse Lipe | Forward | GGGAGGGCCTCAGCG |
|  | Reverse | AGGCCATATTGTCTTCTGCGA |
| Mouse Gpd1l | Forward | GACCACCATTGGCAGCAAAG |
|  | Reverse | CACGGTGTCTGCATCGTCTA |
| Mouse Gk5 | Forward | CTCGCTGTTGGATTTTGGGC |
|  | Reverse | TCTTAAAATGCCTCGTATTCCTGC |
| Mouse Agk | Forward | GCCTGTCAAGAAGCTCAGGT |
|  | Reverse | AGTTCTGGCTTTGCCTTTGC |
| Mouse Dgka | Forward | ACAAGGCTTTGTTGTGCTGC |
|  | Reverse | AGCCCGAGCTGTTATTGGAG |
| Mouse Acss1 | Forward | CGGTTGGATCACAGGACACA |
|  | Reverse | GTCTCCCAGTAACGACCAGC |
| Mouse Acss2 | Forward | TCCAGATGTCCAGATCTGCTG |
|  | Reverse | GAGTGGGTCCTCAGCATCAC |
| Mouse Acsl1 | Forward | AGCCTCACTGCCCTTTTCT |
|  | Reverse | ATGCAGAATTCATCTGTGCCATC |
| Mouse Dgat1 | Forward | CGACGGCTACTGGGATCTGA |
|  | Reverse | CTCAGGATCAGCATCACCACAC |
| Mouse Gpam | Forward | AGCTTCTAAGTCACCCACACC |
|  | Reverse | TGCTTACTGGTCCTGTATCCTTG |
| Mouse Lpin1 | Forward | CCGGCCTGCTGATGTGTATT |
|  | Reverse | GTGATCGACCACTTCGCAGA |
| Mouse Lpin2 | Forward | TGCTTTACCTAGAAGATAACAGTGA |
|  | Reverse | TGGTTTGAGACAGAATCCCAGG |
| mIrs1-Up (site A) ChIP -2570F | Forward | GTTGTGTGTGTGTGAGGGTATGTG |
| mIrs1-Up (site A) ChIP -2418R | Reverse | TCTGCTCTGAGGTGCCCAAG |
| mIrs1-Pro Krox site (site B) ChIP -614F | Forward | GTCGCCTTTACCTGTTGGGTG |
| mIrs1-Pro Krox site (site B) ChIP -413R | Reverse | AGGCTTGTTTGGGTGGCTAC |
| mIrs1-5UTR Krox site (site C) ChIP 401F | Forward | AGAGGAGGAGGAGGAGAAGGAG |
| mIrs1-5UTR Krox site (site C) ChIP 590 R | Reverse | ATGTAGGGAGGCAGCGATGG |
| mIrs1- Ex1 (site D) ChIP +1767F | Forward | ACCATCTCAACAACCCTCCACC |
| mIrs1-Ex1 (site D) ChIP 1968R | Reverse | TCCTGTTGGTGCTAGGGCTC |
| mActin ChIP-F | Forward | GTCTTTCTTCTGCCGTTCT |
| mActin ChIP-R | Reverse | CACTTATCACCAGCCTCAT |
